# Exercise-based interventions for preventing and treating cancer therapy-related cardiovascular toxicity: a systematic review and meta-analysis

**DOI:** 10.1186/s12872-025-04865-8

**Published:** 2025-06-04

**Authors:** Qun Wang, Zehao Huang, Sek Ying Chair

**Affiliations:** 1 School of Nursing, Shenzhen University, Shenzhen, China; 2 The Nethersole School of Nursing, Faculty of Medicine, The Chinese University of Hong Kong, Hong Kong, China

**Keywords:** Cancer therapy, Cardiovascular toxicity, Exercises, Meta-analysis, Systematic review

## Abstract

**Purpose:**

This review aimed to evaluate the effects of exercise-based interventions on cancer therapy-related cardiovascular toxicity (CTR-CVT) in individuals with cancer.

**Methods:**

Four databases (MEDLINE, Embase, Web of Science, and CENTRAL) were searched to identify eligible studies. Randomized controlled trials examining the effects of exercise-based interventions on CTR-CVT in cancer patients published in English were included. The risk of bias of included studies was assessed using version 2 of the Cochrane risk-of-bias tool for randomized trials. The meta-analysis was performed using statistical software R. The PRISMA statement was followed.

**Results:**

Thirty studies with 2484 participants were included. Our findings revealed that compared to the control group, exercise-based intervention improved VO_2peak_ (mean difference [MD]: 1.62, 95% confidence interval [CI]: 0.94 to 2.30), resting diastolic blood pressure (MD: -4.43, 95% CI: -8.72 to -0.13), and resting heart rate (MD=-3.74, 95% CI: -6.59, -0.89) among individuals with cancer. Evidence on other study outcomes remains unclear.

**Conclusion:**

The findings of this review demonstrate the potential role of exercises in preventing and treating CTR-CVT. Further research is warranted to strengthen the current evidence and fill the gaps identified in this review.

**Registration:**

The review protocol was registered in PROSPERO (ID: CRD42022380550).

**Supplementary Information:**

The online version contains supplementary material available at 10.1186/s12872-025-04865-8.

## Introduction

With the advances in cancer treatment and care, the number of cancer survivors is growing [1]. The 5-year relative survival rate for all cancers was 69% from 2014 to 2020 [2]. In the United States, it is estimated that there are more than 18 million cancer survivors by January 2022, and the number of cancer survivors is projected to increase to 26 million by 2040 [3]. The improved prognosis has led to the growing need to address the side effects induced by cancer therapies [4]. Cardiovascular toxicity is one of the most common side effects of cancer therapies. According to the consensus statement of the International Cardio-Oncology Society, cancer therapy-related cardiovascular toxicity (CTR-CVT) encompasses five key categories of cardiovascular events caused by cancer treatments, including cardiac dysfunction, myocarditis, vascular toxicity, hypertension, and arrhythmias and QTc prolongation [5]. Patients undergoing cancer treatments such as chemotherapy, targeted therapy, immune therapies, and radiotherapy are at higher risk of developing cardiovascular toxicity. A recent cohort study showed that the cumulative incidence of CTR-CVT was 71.8% among individuals with breast cancer [6]. Cardiovascular mortality has become the dominant cause of death during cancer survivorship [1, 7]. Therefore, the prevention and treatment of CTR-CVT are of paramount importance.

Currently, several strategies have been adopted to prevent and mitigate CTR-CVT, including reducing cancer drug dosage, optimizing treatment regimens, and administrating cardioprotective drugs. However, the dose adjustments and prescription of cardioprotective drugs may interfere with the cancer treatment effects and produce side effects [8, 9]. Physical exercise is regarded as a promising non-pharmacological therapy to prevent and mitigate CTR-CVT for cancer patients. Combining exercise prescriptions with current CTR-CVT management strategies may show more benefits in improving cancer patient outcomes [1, 10].

The exercise prescriptions for cancer patients typically include endurance and resistance training [10]. It is well documented that exercise-based interventions are safe and effective in improving physical function, psychological distress, quality of life, healthcare services utilization, and mortality among cancer patients [11–14]. Nevertheless, research evidence on the effects of exercises on CTR-CVT among cancer patients is limited. A systematic review of eight studies found that exercises (aerobic continuous, aerobic interval, and/or resistance) may maintain or improve cardiorespiratory fitness in breast cancer patients undergoing chemotherapy, but the evidence for the improvements in left ventricular ejection fraction (LVEF) and global longitudinal strain (GLS) is unclear. This review is limited by the methodological deficiencies of the included studies, and only half of the studies adopted a randomized controlled trial (RCT) design [15]. Similar findings were also reported in the systematic review and meta-analysis conducted by Ma and colleagues [8]. The results of this study indicated that aerobic exercises exerted beneficial effects on VO_2peak_ and the ratio of peak early to late diastolic filling velocity (E/A) among breast cancer patients undergoing anthracycline or trastuzumab treatment. However, this study included limited data (four RCTs, two quasi-experimental controlled trials, and one single group study) with obvious heterogeneity, which diminished the rigorousness [8]. The significant heterogeneity of the synthesized results was also found in the study conducted by Amin et al. [16]. Fakhraei et al. reported that cardiac rehabilitation could improve CRF among cancer survivors in their recent work, but the evidence is inadequate (one RCT and nine retrospective cohort studies) [17]. In another study, evidence on the role of aerobic exercise in protecting against anthracycline-induced cardiotoxicity was also insufficient, as only three clinical studies with small sample sizes were included [18]. Furthermore, recent reviews mainly focus on the effects of exercises on cancer therapy-related cardiac dysfunction, while evidence for other CTR-CVT components is scarce [19–21].

Therefore, this review aimed to systematically evaluate RCTs that evaluated exercise-based interventions’ effects on all CTR-CVT components among cancer patients.

## Methods

We implemented this systematic review and meta-analysis in compliance with the Preferred Reporting Items for Systematic Reviews and Meta-Analyses (PRISMA) Checklist [22]. The review protocol was registered in PROSPERO (ID: CRD42022380550).

### Search strategy

We performed electronic searches for research articles about exercise-based interventions for CTR-CVT in MEDLINE, Embase, Web of Science, and CENTRAL. The search was initially conducted in October 2022 and then updated in March 2025 using the following mesh subject heading and free-text terms: “exercise”, “cancer therapy”, “cardiotoxicity”, and “randomized controlled trial” (Appendix 1). We also identified further eligible studies by handsearching reference lists of included studies and previously published reviews.

### Eligibility criteria

The included studies need to meet the following criteria: (1) adults with a clinical diagnosis of cancer; (2) patients in the experimental group received exercise-based interventions with or without usual care; (3) patients in the control group received various counterpart interventions such as placebo, attention controls, wait-list controls, or usual care; (4) outcomes of interest included LVEF, GLS, cardiac output, stroke volume, the ratio of early diastolic mitral inflow velocity to mitral annular velocity (E/e’), E/A, VO_2peak_, VO_2max_, N-terminal pro B-type natriuretic peptide (NT-proBNP), B-type natriuretic peptide (BNP), high-sensitivity cardiac troponin I (hs-cTnI); high-sensitivity cardiac troponin T (hs-cTnT), high-sensitivity-C-reactive protein (hs-CRP), vascular reactivity (flow-mediated dilation [FMD] and brachial–ankle pulse wave velocity [baPWV]), blood pressure, and heart rate.; (5) randomized controlled trials. Editorials, letters, comments, articles without full text, and publications in non-English languages were excluded.

### Study selection and data extraction

We used Endnote 20 software to manage the records obtained. Two reviewers (QW and ZHH) independently screened the titles, abstracts, and full text of the remaining records in accordance with the eligible criteria. Disagreements were discussed to achieve concordance. Otherwise, a third reviewer (SYC) was consulted. One reviewer (ZHH) conducted the initial data extraction of the details of the eligible studies using a standardized data extraction form, and then a second reviewer (QW) checked the study details. Any discrepancies were discussed to reach a consensus or settled by a third reviewer (SYC). The extracted information included publication information (first author, year of publication, and country), characteristics of the participants (diagnosis, cancer therapy, sample size and age), details of exercise-based interventions (content, number, length, and frequency of session, format, delivery mode, setting, duration, follow-up, and attrition rate), controls, outcomes, and findings.

### Study appraisal

The risk of bias in the eligible studies was assessed independently by two reviewers (QW and ZHH) using version 2 of the Cochrane risk-of-bias tool for randomized trials (RoB 2.0) [23]. All uncertainties were resolved through discussion, with a third reviewer (SYC) acting as an arbiter. The tool is used to evaluate the risk of bias in five domains, including the randomization process, deviations from intended intervention, missing outcome data, measurement of the outcome, and selection of the reported result. Each domain is rated as “high risk of bias”, “low risk of bias”, or “some concerns”. The overall risk of bias in included studies is generated finally in terms of the results of each domain.

### Therapeutic quality of exercise-based interventions

The therapeutic quality of the exercise-based interventions was assessed using the i-CONTENT tool, which included seven items: patient selection, dosage of the exercise program, type of exercise program, qualified supervisor, type and timing of outcome assessment, safety of the exercise program, and adherence to the exercise program. Each item was rated as “low risk of effectiveness”, “high risk of effectiveness”, “probably (done)” or “probably not (done)” [24].

### Statistical analysis

Immediate post-intervention data was used for data synthesis using statistic software R. The mean difference (MD) and standardized mean difference (SMD) with a 95% confidence interval (CI) were calculated to estimate the effect sizes of study outcomes. SMDs of 0.2, 0.5, and 0.8 were interpreted as small, moderate, and large effect sizes, respectively. For eligible studies reporting 95% CI, the standard deviation (SD) was obtained using the following formula: SD=$\sqrt {\rm{N}} {\rm{ \times }}\left( {{\rm{upper}}\:{\rm{limit - lower}}\:{\rm{limit}}} \right){\rm{/3}}{\rm{.92}}$ [25]. The significance level was set at *p* < 0.05. We used MD with 95% CI to calculate the intervention effects on LVEF, VO_2peak_, resting systolic blood pressure (SBP), resting diastolic blood pressure (DBP), mean arterial pressure (MAP), and resting heart rate (HR). For stroke volume, we employed SMD with 95% CI for stroke volume. Heterogeneity within included studies was assessed using the Q test and *I*^*2*^ statistics. Statistical heterogeneity was determined when the *p*-value of the Q test was less than 0.1. *I*^*2*^ values of 0–40%, 30–60%, 50–90%, or 75–100% indicated not important, moderate, substantial, or considerable heterogeneity [25]. Considering clinical and methodological heterogeneity within the included studies, the random-effects model was adopted [25, 26]. We performed subgroup analyses in terms of exercise type for VO_2peak_. Sensitivity analyses were applied to evaluate the robustness of our results by sequential omission of individual studies [27]. We evaluated the publication bias of indicators that included at least ten studies by visually inspecting the contour-enhanced funnel plot [28].

### Certainty of the evidence

The certainties of the evidence for the outcomes were determined in five aspects (risk of bias, inconsistency, indirectness, imprecision, and publication bias) of the Grading of Recommendations Assessment, Development and Evaluation (GRADE), which classified the certainty of the evidence into four grades (very low, low, moderate, and high). We adopted the Grading of Recommendations Assessment, Development and Evaluation profiler Guideline Development Tool (GRADEpro GDT) to produce the summarized findings [25].

## Results

### Search results

As shown in Appendix 2, we extracted 505 records from four databases and five additional records in the initial search. Finally, a total of 30 studies were included in this review. The reasons for the exclusion of ineligible reports included conference abstracts, inappropriate participants and interventions, articles without full text, non-RCT, and outcomes not of interest.

### Characteristics of the included studies

All included studies were published between the years 2004 and 2024. The studies were implemented in the USA (*n* = 7), Canada (*n* = 6), Australia (*n* = 2), China (*n* = 3), Portugal (*n* = 2), Sweden (*n* = 2), France (*n* = 1), Norway (*n* = 1), Poland (*n* = 1), South Korea (*n* = 1), Spain (*n* = 1), Switzerland (*n* = 1), Thailand (*n* = 1) and UK (*n* = 1).

A total of 2484 cancer patients were enrolled in this review, with 1266 in the experimental group and 1218 in the control group. Most studies (20/30) recruited breast cancer patients only. The reported cancer therapies included chemotherapy, radiotherapy, hormone therapy, and targeted therapy.

The prescribed exercises encompassed aerobic exercise, interval training, resistance exercise, and a combination of these exercises. Many included studies (17/30) adopted three weekly intervention sessions. The length of each session varied across different exercise prescriptions and intervention periods. The majority (24/30) conducted interventions with individuals, and nearly all the studies delivered interventions via the face-to-face approach. All but three studies reported the intervention settings and most interventions were implemented in the hospitals (19/30). The intervention contents of the control groups mainly included placebo, usual care, wait-list control, and attention control. The reported intervention duration ranged from one month [29] to 12 months [30]. Moreover, the reported attrition rates varied from 0% [31–33] to 31.1% [34]. The detailed characteristics of the eligible articles are summarized in Appendix 3.

### Quality appraisal of the included studies and i-CONTENT assessment

The results of the quality appraisal are presented in Appendix 4. In terms of the overall risk of bias, three studies were evaluated to have high risks of bias, seven studies were judged as low risks of bias, and the remaining studies were rated to have some concerns about the risks of bias. With respect to specific domains of ROB 2.0, three have high risks of bias in the deviations from the intended intervention domain [34–36]. Ansund et al. [34] and Mijwel et al. [36] failed to implement the protocol interventions, which could affect the intervention effects. Moreover, Ansund et al. [34] and Hojan et al. [35] did not use intention-to-treat analysis and thus had potential impacts on the results. Six studies have some concerns about the risk of bias in the randomization process [34, 37–41]. One study was reported to have a statistically significant difference in one key baseline characteristic between groups [34], and the other studies did not provide sufficient information on the strategies used to achieve randomization and/or allocation concealment [37–41]. We judged seven studies as some concerns in the deviations from intended intervention domain, as all these studies used per-protocol analyses, but it did not cause substantial impact on the results [29, 38, 40–44]. Most studies (20/30) were assessed to have some concerns about the risk of bias in the selection of the reported result domain since there were no protocols available for comparison with the published reports.

In the i-CONTENT assessment, patient selection, dosage of the exercise program, type of the exercise program, and type and timing of outcome assessment were regarded as low risk of ineffectiveness across all the exercise-based interventions. For the qualified supervisor, 47.2% of the exercise-based interventions were rated as “low risk,” and the remaining was classified as “probably done”. Regarding the safety of the exercise program, 58.3% were rated as “low risk”, 38.9% as “probably done”, and one was classified as “high risk”. Concerning the adherence to the exercise program, most of the interventions (61.1%) were rated as “low risk”, 27.8% as “probably done”, and 11.1% as “high risk” (Appendix 5).

### Effects of exercise-based interventions for preventing and treating cancer therapy-related cardiovascular toxicity

#### LVEF

**Fig. 1:**
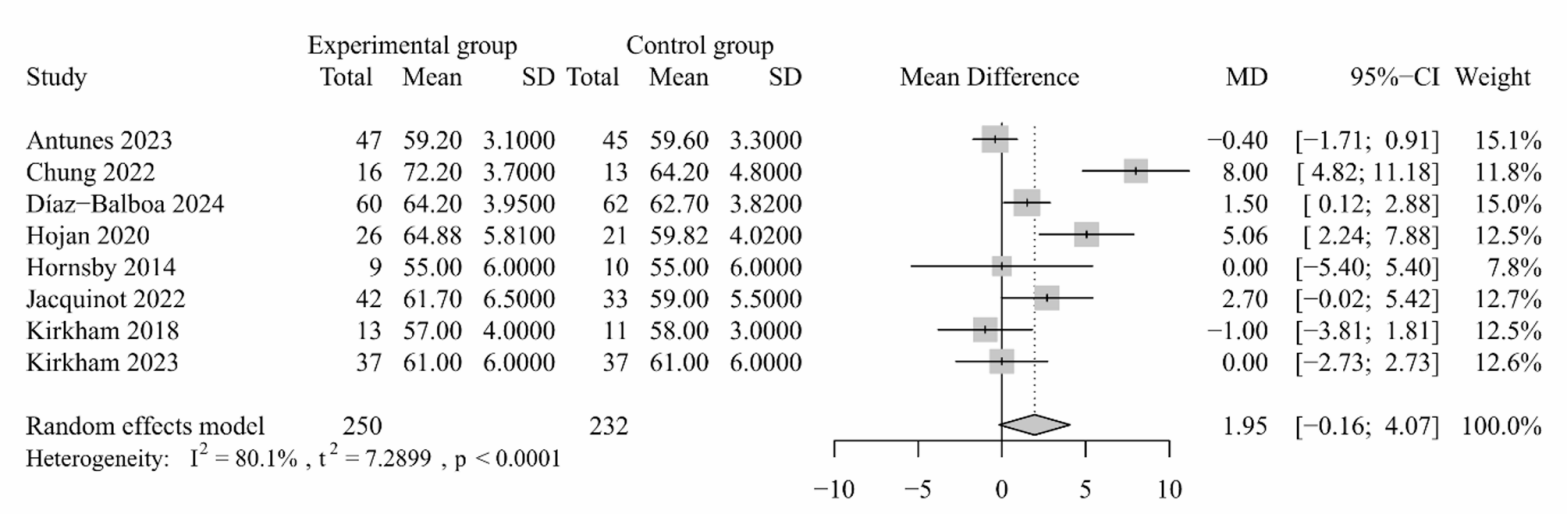
Forest plot for the effect of exercise-based interventions on LVEF

The LVEF was reported in ten included studies [30, 35, 39, 43, 45–50]. Two studies that did not provide raw data found no statistically significant effect of exercise on LVEF [30, 50]. The synthesized results also indicated that there was no significant difference in LVEF between the study groups (eight trials; MD: 1.95, 95% CI: -0.16 to 4.07, *P* = 0.070, *I*^*2*^ = 80.1%; Fig. 1), and these findings remained consistent after sensitivity analysis (Appendix 6).

#### GLS

The effects of exercise-based interventions on GLS were evaluated in eight studies [30, 35, 39, 42, 43, 47, 49, 50]. We did not conduct data synthesis due to the lack of robustness in the pooled results. All included studies showed that exercise-based interventions had no significant effects on GLS.

#### Cardiac output and stroke volume

**Fig. 2:**
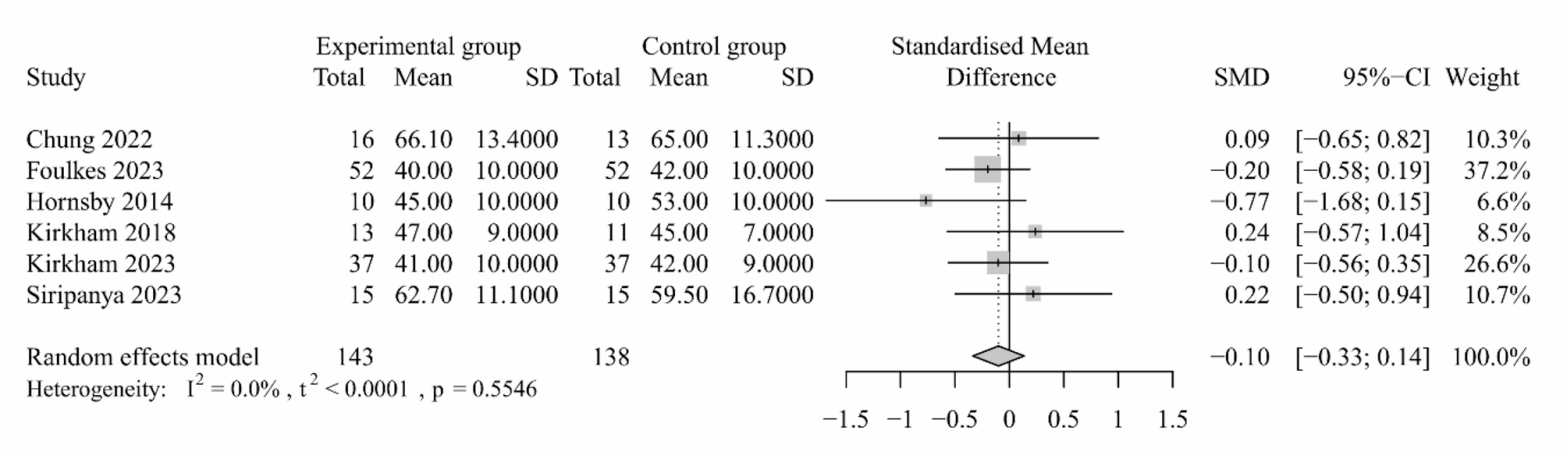
Forest plot for the effect of exercise-based interventions on stroke volume

Three studies reported the effects of exercises on cardiac output and six studies reported stroke volume [30, 41, 43, 45, 47, 48]. We did not combine the studies reporting cardiac output, as the sensitivity analysis indicated the results were not robust. The included studies revealed no significant effects of exercise-based interventions on cardiac output [41, 43, 45]. The meta-analysis indicated that exercise-based interventions exerted no favorable effects on stroke volume among patients with cancer (six trials; SMD: -0.10, 95% CI: -0.33, 0.14, *P* = 0.406, *I*^*2*^ = 0%; Fig. 2) and the pooled results did not alter following sensitivity analysis (Appendix 7).

#### E/A ratio and E/e’ ratio

The E/A ratio was reported in four studies [30, 35, 48, 50]. Of these, only one study showed statistically significant differences between study groups [30]. The findings were not synthesized, as only two studies provided raw data. Two studies also reported a non-significant effect of exercise on the E/e’ ratio between the study groups [30, 50].

#### VO_2peak_ and VO_2max_

**Fig. 3:**
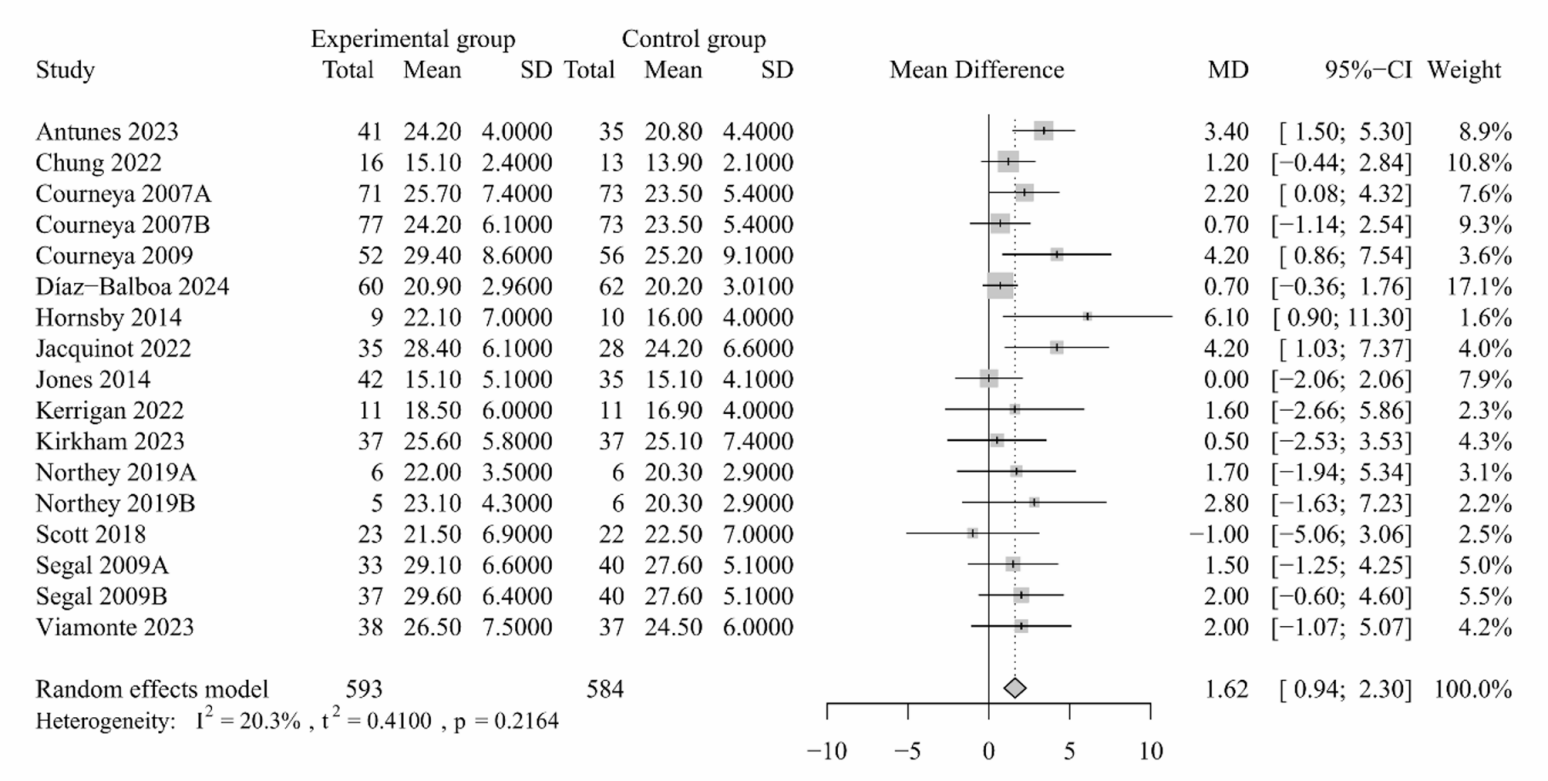
Forest plot for the effect of exercise-based interventions on VO_2peak_

In the studies that reported VO_2peak_ in mL/kg/min, five studies that did not provide raw data or whose data were unsuitable for synthesis revealed the beneficial effects of exercise on VO_2peak_ [30, 36, 40, 41, 50]. Seventeen trials, including 1177 subjects, provided available data for the synthesis to evaluate the effects of exercise-based interventions on VO_2peak_ among cancer patients [31, 39, 42, 44–49, 51–55]. Compared to the controls, the VO_2peak_ was significantly improved in the exercise-based intervention groups, with an MD of 1.62 (95% CI: 0.94 to 2.30, *P* < 0.001, *I*^*2*^ = 20.3%; Fig. 3; 17 trials). The subgroup analysis in terms of exercise type showed significant improvements in aerobic and resistance exercise (MD: 1.48, 95% CI: 0.37 to 2.59; Appendix 8) and aerobic exercise only (MD: 2.21, 95% CI: 0.82 to 3.60; Appendix 8). The combined MD was not altered after sensitivity analysis, indicating the robustness of the result (Appendix 9). Furthermore, no potential publication bias was detected, as the missing studies fell in both the significant and non-significant regions of the funnel plot (Appendix 10). Moreover, two studies reported the effects of exercise-based interventions on VO_2max_ [32, 56], but only one study demonstrated that participants in the experimental groups had better VO_2max_ than the control group [56].

#### NT-proBNP and BNP

Six trials evaluated NT-proBNP among patients with cancer, but only two provided raw data [34, 39, 43, 46, 50]. One study found that a four-month exercise program resulted in lower NT-proBNP in the exercise group at 12 months [34]. Furthermore, an included study assessed BNP and found that exercise had no effect on BNP among patients with cancer [47].

#### hs-cTnT and hs-cTnI

The hs-cTnT was reported in five trials; however, only two studies provided raw data. All studies demonstrated no statistically significant differences between groups [34, 43, 46, 50]. In addition, four studies measured hs-cTnI [30, 39, 42, 47], with only one study reporting that exercise improved hs-cTnI among patients with cancer [30].

#### hs-CRP

Four studies examined the effect of exercise-based interventions on hs-CRP and raw data were only available from two studies [35, 41, 44, 50]. Among these, a home-based Buddhist walking program showed beneficial effects on improving hs-CRP [41].

#### FMD and BaPWV

In the study of Siripanya et al. [41], the authors observed a significant effect of home-based Buddhist walking on FMD among patients with cancer. Nevertheless, Jones et al. [57] revealed no significant between-group difference in FMD. With regard to baPWV, a favorable effect of exercise was identified in the study by Lee et al. [33], whereas Siripanya et al. [41] found no significant differences between groups.

#### Blood pressure

**Fig. 4:**
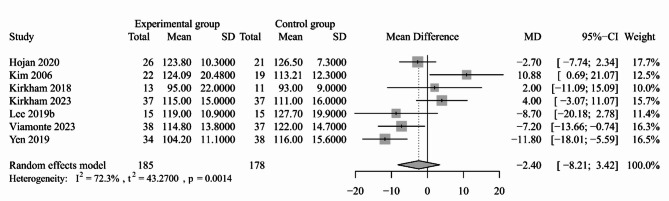
Forest plot for the effect of exercise-based interventions on resting SBP

**Fig. 5:**
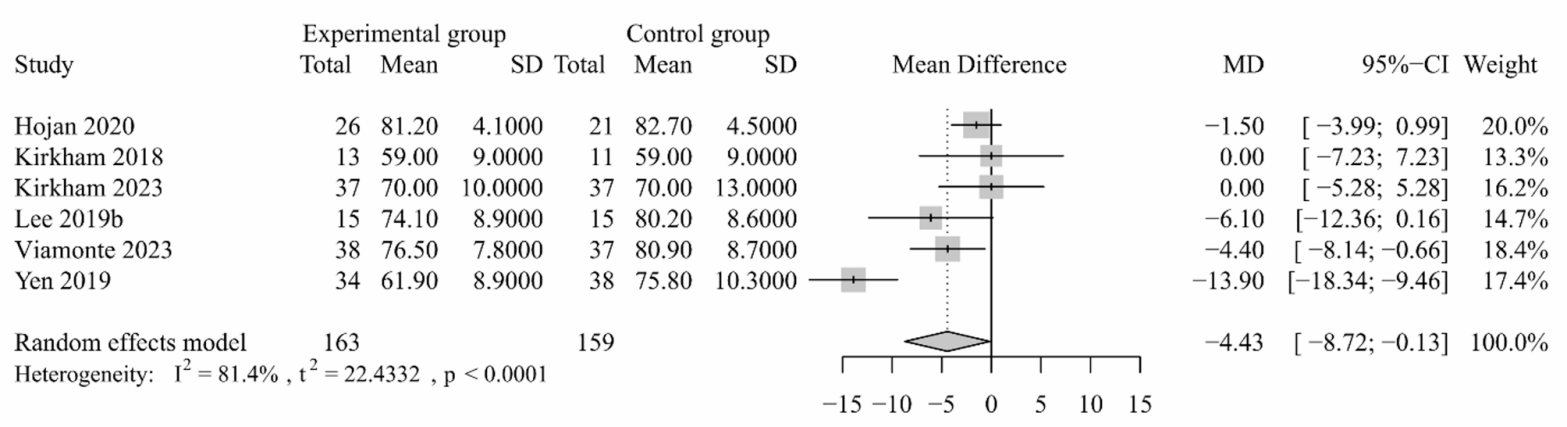
Forest plot for the effect of exercise-based interventions on resting DBP

**Fig. 6:**
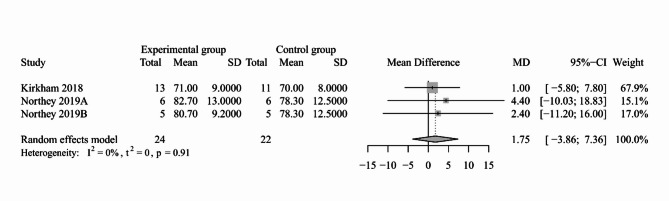
Forest plot for the effect of exercise-based interventions on MAP

Resting SBP was measured in eight studies [33, 35, 37, 38, 43, 44, 47, 50]. The results of meta-analysis showed that there was no significant difference in resting SBP between the study groups (seven trials; MD: -2.40, 95% CI: -8.21 to 3.42, *P* = 0.420, *I*^*2*^ = 72.3%; Fig. 4), and this finding remained consistent after sensitivity analysis (Appendix 11). Seven studies evaluated the effects of exercises on resting DBP [33, 35, 38, 43, 44, 47, 50]. Current evidence showed a significant effect of exercise-based interventions on resting DBP (six trials; MD: -4.43, 95% CI: -8.72 to -0.13, *P* = 0.043, *I*^*2*^ = 81.4%; Fig. 5), which was supported by sensitivity analysis (Appendix 12). One study that did not provide raw data on SBP and DBP also found a non-significant effect of exercise on these outcomes [50]. The peak SBP and DBP were measured in two studies [30, 38], but no significant between-group differences were found. Three trials assessed the impact of exercise-based interventions on MAP [31, 43]. The meta-analysis suggested that exercise-based interventions had no significant effect on improving MAP in cancer patients (three trials; MD: 1.75, 95% CI: -3.86 to 7.36, *P* = 0.54, *I*^*2*^ = 0%; Fig. 6), and this result was reliable as the pooled MD was not changed after sensitivity analysis (Appendix 13).

#### Heart rate

**Fig. 7:**
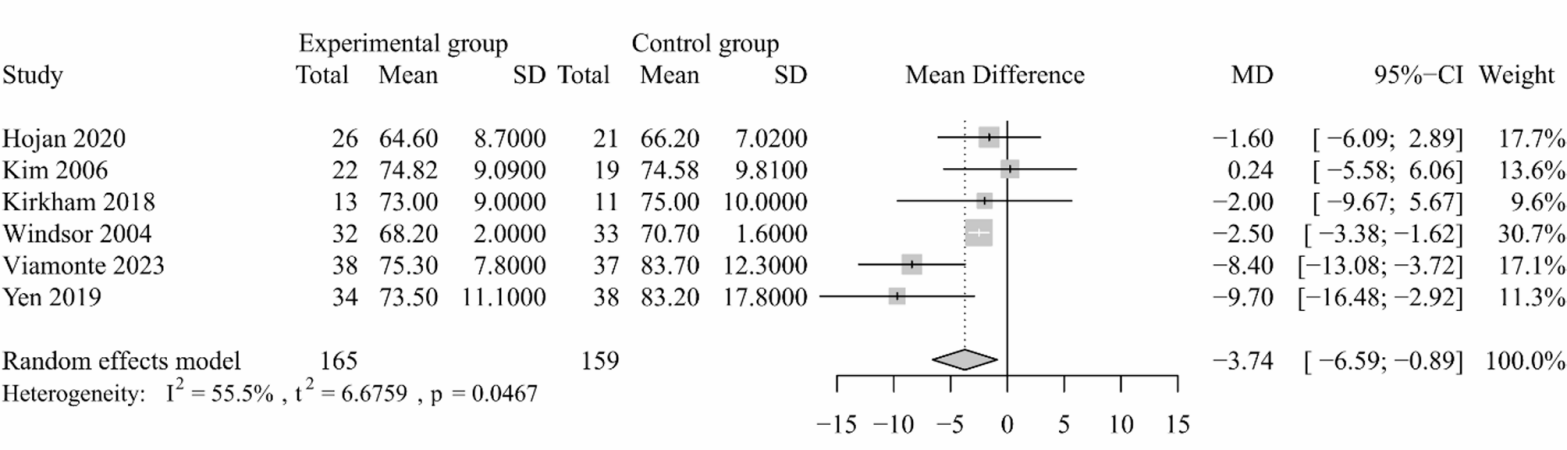
Forest plot for the effect of exercise-based interventions on resting HR

The resting HR was reported in six included studies [29, 35, 37, 38, 43, 44]. The meta-analysis showed that exercise-based interventions had a significant effect on resting HR among patients with cancer (six trials; MD: -3.74, 95% CI: -6.59, -0.89, *P* = 0.010, *I*^*2*^ = 55.5%; Fig. 7) and this result remained unchanged following sensitivity analysis (Appendix 14). Six studies reported the effects of exercise-based intervention on peak HR [30, 38, 42, 48, 49, 52]. We did not synthesize the data considering the substantial heterogeneity within trials and the results were not reliable based on the sensitivity analysis. Among these studies, only one study reported a statistically significant finding [38]. Heart rate variability (HRV) was evaluated in one study, which indicated that compared with controls, exercise-based interventions could improve HRV in cancer patients [58].

### Certainty of the evidence

The level of evidence for VO_2peak_ and NT-proBNP was rated as high. Resting DBP was rated as low because of significant heterogeneity and limited sample size. The other outcomes were assigned a moderate rating due to limited sample sizes and/or narrative synthesis (Appendix 15).

## Discussion

In this study, we summarized the available evidence on the effects of exercise-based interventions on CTR-CVT among cancer patients. The meta-analyses demonstrated that exercises could improve VO_2peak_, resting DBP, MAP, and resting HR when compared to the controls, while robust evidence on other study outcomes remains unclear. The findings of this study indicated that exercise-based interventions had potential benefits in preventing and managing CTR-CVT.

Echocardiography-related outcomes provide insights into the cardiac function of individuals with cancer [59]. Current guidelines highlight the importance of evaluating LVEF and GLS for the early detection and diagnosis of CTR-CVT [1, 5]. In line with a previous study [16, 21], our review found no supporting evidence of the favorable effects of exercise-based interventions on these clinical outcomes. Existing evidence indicates that a combination of aerobic exercises and resistance training may be effective in improving LVEF, as positive results were only found in the study conducted by Hojan et al. [35], Díaz-Balboa et al. [39], and Chung et al. [48]. Future studies may consider using three-dimensional echocardiography to assess LVEF, as it is superior to the two-dimensional echocardiography currently adopted in the included studies [60]. In addition, this review did not identify positive effects of exercise-based interventions on GLS in patients with cancer. Therefore, it is suggested that more trials are needed to investigate the effects of appropriate exercise regimes on LVEF and GLS among cancer patients. This review also summarized available data for the effects of exercise-based interventions on cardiac output, stroke volume, E/A ratio, and E/e’ among patients with cancer. A 12-month multimodal exercise program that integrated aerobic exercise, resistance training, and high-intensity interval training demonstrated positive effects on E/A ratio [30]. However, eligible studies are limited, indicating that further research is necessary.

Cardiorespiratory fitness reflects an individual’s functional capacity and cardiovascular health [61]. Poor cardiorespiratory fitness can affect cardiac function and have higher risk of developing cardiovascular diseases, leading to a higher CTR-CVT prevalence [1, 62]. The findings of our study provide evidence of the beneficial effects of exercise-based interventions on cardiorespiratory fitness in cancer patients, which is consistent with previously published reviews [8, 15, 16, 21]. Notably, we included a greater number of eligible studies and a larger sample size in the meta-analysis, with lower heterogeneity among included trials. Moreover, the sensitivity analysis confirmed the robustness of the evidence.

The importance of cardiac serum biomarkers (e.g., hs-cTnI, hs-cTnT, BNP, NT-proBNP, hs-CRP) for monitoring CTR-CVT has been documented in the current guidelines [1, 5]. However, our review did not provide sufficient evidence of the beneficial effects of exercises on these indicators due to limited available data. Hence, more studies are required to assess the impact of exercise-based interventions on these outcomes. Among the included studies, we observed significant improvements in NT-proBNP among patients with cancer following a combination of resistance and high-intensity interval training, or a combination of aerobic exercise and high-intensity interval training at a longer follow-up period [34]. Furthermore, a 12-month multimodal exercise program that integrated aerobic exercise, resistance training, and high-intensity interval training demonstrated positive effects on hs-cTnI [30]. In addition, a home-based Buddhist walking program was related to lower hs-CRP [41].

Vascular toxicity of cancer therapies often results from endothelial dysfunction [63]. It may negatively affect arterial elasticity and ultimately lead to cardiovascular diseases [64]. Nevertheless, the present study does not provide sufficient evidence regarding the effects of exercise-based interventions on vascular toxicity, suggesting that more studies are required before a robust conclusion can be drawn. Among the eligible trials, a significant impact of exercise on FMD was noted in a 12-week home-based Buddhist walking program. Additionally, a 2-month high-intensity interval training program positively affected baPWV [33].

Hypertension, a significant predictor for a variety of cardiovascular diseases, is well-known as one of the main types of CTR-CVT in cancer patients [1]. In our review, we can only provide limited evidence supporting the effects of exercise-based interventions on resting DBP among patients with cancer. The favorable effects on resting SBP and peak SBP and DBP cannot be identified in our study. Moreover, the meta-analysis of the three included trials suggests that exercises are not able to exert beneficial effects on MAP. Therefore, future research to reveal the role of exercise in blood pressure is therefore essential.

It is well-known that resting HR is a crucial indicator of cardiovascular diseases and long-term mortality in cancer patients, and patients with higher levels of resting HR usually have higher levels of cardiac biomarkers and an increased risk of new-onset atrial fibrillation [65, 66]. Our findings showed that exercise-based interventions can have beneficial effects on resting HR among patients with cancer, which contradicts previous evidence [16]. This discrepancy may be due to the limited number of trials included and the smaller sample sizes in the previous study [16]. We also summarized the available data on the effects of exercises on peak HR, and only an aerobic and resistance exercise program implemented by Yen et al. [38] revealed beneficial effects. Moreover, it has been demonstrated that HRV is a crucial factor in cardiovascular health [67]. In this review, only one five-weekly Tai Chi intervention reported the measurement of HRV and revealed positive findings [58]. Further studies are required to provide more available evidence on the impact of exercises on HR.

Several limitations need to be acknowledged. First, the effects on some outcomes of interest cannot be determined due to the limited included studies for each outcome and the lack of robustness of the pooled results. Second, only seven included studies were assessed to have low risks of bias. Last, most studies were conducted in patients with breast cancer and there is little evidence for other cancer types. Further rigorous studies on different types of cancer are required to provide more available evidence.

## Conclusion

This review suggests that exercise-based interventions have the potential to prevent and treat CTR-CVT among cancer patients. Future well-designed RCTs are needed to evaluate the impact of exercises on CTR-CVT.

## Electronic supplementary material

Below is the link to the electronic supplementary material.

Supplementary Material 1

Supplementary Material 2

Supplementary Material 3

## Data Availability

Data cannot be shared openly but are available on request from authors.

## Abbreviations

CTR-CVT: Cancer therapy-related cardiovascular toxicity
LVEF: Left ventricular ejection fraction
GLS: Global longitudinal strain
E/A: Peak early to late diastolic filling velocity
E/e': Early diastolic mitral inflow velocity to mitral annular velocity
NT-proBNP: N-terminal pro B-type natriuretic peptide
BNP: B-type natriuretic peptide
hs-CRP: hs-C-reactive protein
FMD: Flow-mediated dilation
baPWV: Brachial–ankle pulse wave velocity
MD: Mean difference
SMD: Standardized mean difference
SBP: Systolic blood pressure
DBP: Diastolic blood pressure
MAP: Mean arterial pressure
HR: Heart rate
HRV: Heart rate variability
