# Supplementary material for: Exercise-based interventions for preventing and treating cancer therapy-related cardiovascular toxicity: a systematic review and meta-analysis
Source: BMC Cardiovasc Disord. 2025 Jun 4;25:433. doi: 10.1186/s12872-025-04865-8 (PMC12135320; doi:10.1186/s12872-025-04865-8)
Supplement: Supplementary file 3 — Supplementary Material 3 [file 12872_2025_4865_MOESM3_ESM.docx]

Appendix 1 Search strategy

| Databases | Details |
| --- | --- |
| MEDILINE/PubMed (33) | ("Exercise"[MeSH Terms] OR "Exercise Therapy"[MeSH Terms] OR "Rehabilitation"[MeSH Terms] OR ("exercise*"[Title/Abstract] OR "exercise therap*"[Title/Abstract] OR "rehabilitation*"[Title/Abstract] OR "exercise training*"[Title/Abstract] OR "remedial exercise*"[Title/Abstract] OR "physical activit*"[Title/Abstract] OR "rehabilitation exercise*"[Title/Abstract] OR "aerobic exercise*"[Title/Abstract] OR "high intensity interval training*"[Title/Abstract] OR "resistance training*"[Title/Abstract] OR "muscle stretching exercise*"[Title/Abstract])) AND ("Neoplasms"[MeSH Terms] OR ("neoplasm*"[Title/Abstract] OR "cancer*"[Title/Abstract] OR "tumor*"[Title/Abstract] OR "malignant neoplasm*"[Title/Abstract] OR "benign neoplasm*"[Title/Abstract]) OR ("Drug Therapy"[MeSH Terms] OR "Immunotherapy"[MeSH Terms] OR "Radiotherapy"[MeSH Terms] OR "Stem Cell Transplantation"[MeSH Terms] OR ("drug therap*"[Title/Abstract] OR "pharmacotherap*"[Title/Abstract] OR "immunotherap*"[Title/Abstract] OR "radiotherap*"[Title/Abstract] OR "stem cell transplantation*"[Title/Abstract] OR "chemotherap*"[Title/Abstract] OR "targeted agent*"[Title/Abstract] OR "targeted therap*"[Title/Abstract] OR "hormone therap*"[Title/Abstract] OR "cancer therap*"[Title/Abstract] OR "cancer treatment*"[Title/Abstract]))) AND ("Cardiotoxicity"[MeSH Terms] OR ("cardiotoxicit*"[Title/Abstract] OR "cardiac toxicit*"[Title/Abstract] OR "cardiovascular toxicit*"[Title/Abstract])) AND ("Randomized Controlled Trial"[Publication Type] OR "randomized controlled trial*"[Title/Abstract]) |
| Embase (206) | 1 exp exercise/ or exercise*.mp.  2 exercise therap*.mp.  3 rehabilitation*.mp.  4 exercise training*.mp.  5 remedial exercise*.mp.  6 exp physical activity/ or physical activit*.mp.  7 rehabilitation exercise*.mp.  8 exp aerobic exercise/ or aerobic exercise*.mp.  9 exp high intensity interval training/ or high-intensity interval training*.mp.  10 exp resistance training/ or resistance training*.mp.  11 exp stretching exercise/ or muscle stretching exercise*.mp.  12 1 or 2 or 3 or 4 or 5 or 6 or 7 or 8 or 9 or 10 or 11  13 neoplasm*.mp. or exp neoplasm/  14 cancer*.mp.  15 tumor*.mp.  16 malignant neoplasm*.mp.  17 benign neoplasm*.mp.  18 exp drug therapy/ or drug therap*.mp.  19 exp immunotherapy/ or immunotherap*.mp.  20 pharmacotherap*.mp.  21 exp radiotherapy/ or radiotherap*.mp.  22 exp hematopoietic stem cell transplantation/ or stem cell transplantation*.mp.  23 exp chemotherapy/ or chemotherap*.mp.  24 exp molecularly targeted therapy/ or targeted agent*.mp.  25 exp targeted therapy/ or targeted therap*.mp.  26 exp hormone/ or hormone therap*.mp.  27 13 or 14 or 15 or 16 or 17 or 18 or 19 or 20 or 21 or 22 or 23 or 24 or 25 or 26  28 exp cardiotoxicity/ or cardiotoxicit*.mp.  29 cardiovascular toxicit*.mp.  30 28 or 29  31 randomized controlled trial/ or randomized controlled trial.mp.  32 12 and 27 and 30 and 31 |
| Web of Science (101) | exercise* OR exercise therap* OR rehabilitation* OR exercise training* OR remedial exercise* OR physical activit* OR rehabilitation* OR aerobic exercise* OR high-intensity interval training* OR resistance training* OR muscle stretching exercise* (Topic) AND neoplasm* OR cancer* OR tumor* OR malignant neoplasm* OR benign neoplasm* OR drug therap* OR immunotherap* OR radiotherap* OR pharmacotherap* OR cancer therap* OR cancer treatment* OR stem cell transplantation* OR chemotherap* OR targeted agent* OR targeted therap* OR hormone therap* (Topic) AND cardiotoxicit* OR cardiac toxicit* OR cardiovascular toxicit* (Topic) AND randomized controlled trial* (Topic) |
| CENTRAL (165) | #1 MeSH descriptor: [Exercise] explode all trees  #2 MeSH descriptor: [Exercise Therapy] explode all trees  #3 MeSH descriptor: [Rehabilitation] explode all trees  #4 (exercise*):ti,ab,kw OR (exercise training*):ti,ab,kw OR (remedial exercise*):ti,ab,kw OR (physical activit*):ti,ab,kw OR (rehabilitation exercise*):ti,ab,kw  #5 (aerobic exercise*):ti,ab,kw OR (high-intensity interval training):ti,ab,kw OR (resistance training*):ti,ab,kw OR (muscle stretching exercise*):ti,ab,kw  #6 #1 OR #2 OR #3 OR #4 OR #5  #7 MeSH descriptor: [Neoplasms] explode all trees  #8 MeSH descriptor: [Drug Therapy] explode all trees  #9 MeSH descriptor: [Immunotherapy] explode all trees  #10 MeSH descriptor: [Radiotherapy] explode all trees  #11 MeSH descriptor: [Stem Cell Transplantation] explode all trees  #12 (neoplasm*):ti,ab,kw OR (cancer*):ti,ab,kw OR (tumor*):ti,ab,kw OR (malignant neoplasm*):ti,ab,kw OR (benign neoplasm*):ti,ab,kw  #13 (cancer therap*):ti,ab,kw OR (cancer treatment*):ti,ab,kw OR (drug therap*):ti,ab,kw OR (pharmacotherap*):ti,ab,kw OR (immunotherap*):ti,ab,kw  #14 (radiotherap*):ti,ab,kw OR (stem cell transplantation*):ti,ab,kw OR (chemotherap*):ti,ab,kw OR (targeted agent*):ti,ab,kw OR (targeted therap*):ti,ab,kw  #15 (hormone therap*):ti,ab,kw  #16 # 6 OR # 7 OR #8 OR #9 OR #10 OR #11 OR #12 OR #13 OR #14 OR #15  #17 MeSH descriptor: [Cardiotoxicity] explode all trees  #18 (cardiotoxicit*):ti,ab,kw OR (cardiac toxicit*):ti,ab,kw OR (cardiovascular toxicit*):ti,ab,kw  #19 #17 OR #18  #20 MeSH descriptor: [Randomized Controlled Trial] explode all trees  #21 (randomized controlled trial*):ti,ab,kw  #22 #20 OR #21  #23 #6 AND #16 AND #19 AND #22 |

**Identification of studies via other methods**

**Identification of studies via databases and registers**

Records identified from:

Citation searching (n = 5)

Records removed *before screening*:

Duplicate records removed (n = 128)

Records identified from:

MEDLINE/PubMed (n = 33)

Embase (n = 206)

Web of Science (n= 101)

CENTRAL (n = 165)

**Identification**

Records screened

(n = 377)

Records excluded

(n = 319)

Reports not retrieved

(n = 0)

Reports sought for retrieval

(n = 5)

Reports sought for retrieval

(n = 58)

Reports not retrieved

(n = 0)

**Screening**

Reports excluded:

Conference abstracts (n = 7)

Non-targeted participants (n = 4)

Non-targeted interventions (n = 1)

Articles without full text (n = 3)

Not RCT (n = 6)

Reports assessed for eligibility

(n = 5)

Reports excluded:

Outcomes not of interest (n = 1)

Reports assessed for eligibility

(n = 58)

Studies included in review

(n = 30)

Reports of included studies

(n = 41)

**Included**

Appendix 2 PRISMA flow diagram of study selection

Appendix 3 Characteristics of included studies

| Study/Country | Participants | Experimental group | Control group | Duration/Follow-up (Attrition rate) | Outcomes & Findings |
| --- | --- | --- | --- | --- | --- |
| Ansund et al. (2021)A/Sweden | **Diagnosis:** breast cancer  **Cancer therapy:** chemotherapy (anthracyclines and/or taxanes)  **Sample size:**  N=56  EG=29  CG=27  **Mean age:**  EG=53.5±10.2  CG**=**55.9±7.5 | **Content:** resistance and high-intensity interval training  **No./length/frequency of session:** two weekly exercises (resistance training: 8–12 repetitions at 75–80% of one repetition maximum targeting the major muscle groups; 3×3 min bouts of aerobic high intensity interval training on a cycle ergometer)  **Format/delivery mode:** individual; face to face  **Setting:** hospital | Usual care (written information about the exercise recommendations based on guidelines) | 4 months/Baseline-4-12-24 months (24.4%) | hs-cTnT: no significant difference between groups;  NT-proBNP: significantly lower in the exercise group at 12 months;  VO_2peak_: no significant difference between groups at 12 and 24 months |
| Ansund et al. (2021)B/Sweden | **Diagnosis:** breast cancer  **Cancer therapy:** chemotherapy (anthracyclines and/or taxanes)  **Sample size:**  N=59  EG=32  CG=27  **Mean age:**  EG=53.7±7.9  CG**=**55.9±7.5 | **Content:** moderate-intensity aerobic and high-intensity interval training  **No./length/frequency of session:** two weekly exercises (20-min moderate-intensity continuous aerobic exercise and 3×3 min bouts of aerobic high intensity interval training on a cycle ergometer)  **Format/delivery mode:** individual; face to face  **Setting:** hospital | Usual care (written information about the exercise recommendations based on guidelines) | 4 months/Baseline-4-12-24 months (31.1%) | hs-cTnT: no significant difference between groups;  NT-proBNP: significantly lower in the exercise group at 12 months;  VO_2peak_: no significant difference between groups at 12 and 24 months |
| Antunes et al. (2023)/Portugal | **Diagnosis:** breast cancer  **Cancer therapy:** chemotherapy (anthracyclines)  **Sample size:**  N=93  EG=47  CG=46  **Mean age:**  EG=49.66±9.43  CG**=**51.02±9.54 | **Content:** exercise training program **(**warm up, aerobic and resistance training, and cool down)  **No./length/frequency of session:** three weekly sessions  **Format/delivery mode:** individual; face to face  **Setting:** hospital | Usual care | Not reported/Baseline-end of chemotherapy-3 months after the end of chemotherapy (3.2%) | VO_2peak_: significant difference between groups at both study times points  LVEF, NT-proBNP, and hs-cTnT: no significant difference between groups; |
| Chung et al. (2022)/Taiwan, China | **Diagnosis:** breast cancer  **Cancer therapy:** chemotherapy (anthracyclines)  **Sample size:**  N=29  EG=16  CG=13  **Mean age:**  EG=52.4±8.9  CG**=**50.3±7.7 | **Content:** exercise training (40-min aerobic exercise, 15-min resistance exercise, and 5-min flexibility training)  **No./length/frequency of session:** three weekly sessions  **Format/delivery mode:** individual; face to face  **Setting:** hospital | Usual care | 3 months/Baseline-1.5 months-3 months-6 months-12 months (9.4%) | LVEF: significant difference between groups at 3 months  VO_2peak_, stroke volume, peak HR, and E/A ratio: no significant difference between groups at 3 months |
| Courneya et al. (2007)A/Canada | **Diagnosis:** breast cancer  **Cancer therapy:** chemotherapy (Nontaxane or taxane)  **Sample size:**  N=160  EG=78  CG=82  **Mean age:**  EG= 49.0  CG= 49.0 | **Content:** aerobic exercise training (warm-up and cool down of light aerobic activity and stretching and exercise on a cycle ergometer, treadmill, or elliptical)  **No./length/frequency of session:** three weekly exercises (5-min warm-up, 5-minute cool down; exercise began at 60% of VO_2max_ for weeks 1 to 6, 70% at weeks 7 to 12, and 80% at week 12; exercise duration began at 15 minutes for weeks 1 to 3 and increased by 5 minutes every three weeks until reached 45 minutes at week 18)  **Format/delivery mode:** individual; face to face  **Setting:** hospital | Usual care (1-month exercise program after postintervention assessments) | 17 weeks (9 to 24 weeks)/Baseline-postintervention (not reported) | VO_2peak_: significantly superior in the exercise group |
| Courneya et al. (2007)B/Canada | **Diagnosis:** breast cancer  **Cancer therapy:** chemotherapy (Nontaxane or taxane)  **Sample size:**  N=164  EG=82  CG=82  **Mean age:**  EG=49.5  CG=49.0 | **Content:** resistance exercise training (nine different exercises: leg extension, leg curl, leg press, calf raises, chest press, seated row, triceps extension, biceps curls, and modified curl-ups)  **No./length/frequency of session:** three weekly exercises (2 sets of 8-12 repetitions at 60% to 70% of one repetition maximum; the resistance was increased by 10% when patients completed more than 12 repetitions)  **Format/delivery mode:** individual; face to face  **Setting:** hospital | Usual care (1-month exercise program after postintervention assessments) | 17 weeks (9 to 24 weeks)/Baseline-postintervention (not reported) | VO_2peak_: significantly superior in the exercise group |
| Courneya et al. (2009)/Canada | **Diagnosis:** lymphoma  **Cancer therapy:** chemotherapy  **Sample size:**  N=122  EG=60  CG=62  **Mean age:**  EG=52.8  CG=53.5 | **Content:** aerobic exercise training (upright or recumbent cycle ergometer)  **No./length/frequency of session:** three weekly exercises (Intensity began at 60% of the peak power output, which corresponded with baseline VO_2peak_, and was increased by 5% each week to 75% by the fourth week. Duration began at 15 to 20 minutes for the first 4 weeks and increased by 5 minutes per week to 40 to 45 minutes in the ninth week; one session per week of interval training above the ventilatory threshold in week 7 and one session of VO2peak interval training in week 9)  **Format/delivery mode:** individual; face to face  **Setting:** hospital | Usual care (4 weeks supervised exercise after postintervention assessments) | 12 weeks/Baseline-3-9 months (not reported) | VO_2peak_: significantly superior in the exercise group |
| Díaz-Balboa et al. (2024)/Spain | **Diagnosis:** breast cancer  **Cancer therapy:** adjuvant or neoadjuvant anthracyclines and/or anti-HER2 antibodies  **Sample size:**  N=122  EG=60  CG=62  **Mean age:**  EG=48.82±8.02  CG=48.92±8.51 | **Content:** Exercise-based cardio-oncology rehabilitation (5-minute preparation; 10-minute breathing, flexibility, and body weight strengthening; 10 to 15-minute strengthening with elastic bands and standing; 25 to 30-minute cardiovascular training; 5-minute flexibilization)  **No./length/frequency of session:** two 1-hour weekly exercises  **Format/delivery mode:** individual; face to face on site and online  **Setting:** hospital and home | Usual care (Physical activity advice via telephone every two months with motivational interviewing | Average duration: 5.79 months (8.2%) | LVEF and VO_2peak_: significant differences between groups  GLS, NT-proBNP, and hs-cTnI: no significant difference between groups |
| Dolan et al. (2016)A/Canada | **Diagnosis:** breast cancer  **Cancer therapy:** different combinations of surgery, chemotherapy, radiation, and hormonal therapy  **Sample size:**  N=22  EG=12  CG=10  **Mean age:**  EG=56.2±9.0  CG=59.4±9.0 | **Content:**  aerobic interval training  **No./length/frequency of session:** three weekly exercises (introductory intervals for first two weeks at a maximal intensity of 80% VO_2peak_, followed by progressively higher intensity interval bouts for 4 weeks, eventually requiring 2 min efforts that would elicit close to a maximal effort)  **Format/delivery mode:** individual; face to face  **Setting:** not reported | Delayed exercise intervention | 6 weeks/Baseline-6 weeks (9.1%) | VO_2peak_: significantly improved in the exercise group |
| Dolan et al. (2016)B/Canada | **Diagnosis:** breast cancer  **Cancer therapy:** different combinations of surgery, chemotherapy, radiation, and hormonal therapy  **Sample size:**  N=21  EG=11  CG=10  **Mean age:**  EG=56.3±9.0  CG=59.4±9.0 | **Content:** continuous moderate training  **No./length/frequency of session:** three weekly exercises (completed 3.22 km (2 mi) at an initial intensity of 55% to 60% VO_2peak_; progressed to 4.02 km (2.5 mi) at 70% VO_2peak_ by the end of week 5)  **Format/delivery mode:** individual; face to face  **Setting:** not reported | Delayed exercise intervention | 6 weeks/Baseline-6 weeks (14.3%) | VO_2peak_: significantly improved in the exercise group |
| Foulkes et al. (2023)/Australia | **Diagnosis:** breast cancer  **Cancer therapy:** chemotherapy (anthracycline)  **Sample size:**  N=104  EG=52  CG=52  **Mean age:**  EG=56.3±9.0  CG=59.4±9.0 | **Content:** multimodal exercise intervention (moderate-intensity endurance training, tempo training, high-intensity interval training, and moderate-to-high-intensity resistance training)  **No./length/frequency of session:** phase 1: three weekly sessions during chemotherapy (12-week supervised exercise); Phase 2: four weekly sessions after chemotherapy (14-week semi-supervised exercise); phase 3: four weekly sessions (26-week unsupervised maintenance exercise)  **Format/delivery mode:** individual; face to face and online  **Setting:** | Usual care (guideline-consistent care) | 12 months/baseline-4 months-12 months (16.3%) | VO_2peak_, cardiac output, stroke volume, E/A ratio, and hs-cTnI : significant difference between groups  BNP, LVEF, E/e' ratio, and GLS: no significant difference between groups |
| Hojan et al. (2020)/Poland | **Diagnosis:** breast cancer  **Cancer therapy:** targeted therapy (trastuzumab)  **Sample size:**  N=47  EG=26  CG=21  **Mean age:**  EG=54.44±6.29  CG=54.64±5.26 | **Content:** physical training (endurance exercises (brisk walking, running on a treadmill, and various cycling activities) and resistance exercises (isometric, concentric, and eccentric training in different positions for the trunk, upper body, and leg muscles))  **No./length/frequency of session:** five weekly exercises (45 to 50-min endurance training: 2-min warm-up, 45-min one or two aerobic activities, and 3-min relaxation; 40 to 45-min resistance exercise: one to three sets of 8–10 repetitions of selected exercises)  **Format/delivery mode:** individual (exercises on treadmills or cycle ergometers) and group (resistance training); face to face  **Setting:** hospital (outpatient rehabilitation ward) | Usual care (usual daily activities and printed physical activity instructions) | 9 weeks/ Baseline-9 weeks (27.9%) | LVEF: significantly decreased in the control group;  GLS, E/A ratio, HR, SBP, DBP, and hs-CRP: no significant difference between groups |
| Hornsby et al. (2014)/USA | **Diagnosis:** breast cancer  **Cancer therapy:** Neoadjuvant chemotherapy (doxorubicin and cyclophosphamide)  **Sample size:**  N=20  EG=10  CG=10  **Mean age:**  EG=51±6  CG=46±11 | **Content:** aerobic training (cycle ergometry)  **No./length/frequency of session:** three weekly exercises (15 to 20-min at 60% of baseline peak workload in week 1; 30-min at 65% peak workload in week 2 to 4; 30 to 45-min at 60% to 65% peak workload for two sessions and 20 to 25-min for the remaining session in week 5 and 6; 20 to 30-min at 60% to 70% peak workload from week 7 onwards; 20 to 30-min at 60% to 70% peak workload for two sessions with on interval session at 100% peak workload, which consisted of 30s at peak workload and 60s of active recovery for 10 to 15 intervals)  **Format/delivery mode:** individual; face to face  **Setting:** hospital | Maintain the usual exercise level | 3 months/Baseline-3 months (5%) | VO_2peak_: significantly increased in the exercise group;  Cardiac output, stroke volume, and LVEF: no significant difference between groups |
| Jacquinot et al. (2022)/France | **Diagnosis:** breast cancer  **Cancer therapy:** targeted therapy (trastuzumab)  **Sample size:**  N=89  EG=46  CG=43  **Mean age:**  EG=51.1 [28.7-66.8]  CG=51.0 [32.6-74.5] | **Content:** supervised exercise program (carried out on a cycloergometer, with electromagnetic braking)  **No./length/frequency of session:** three 55-min weekly sessions  **Format/delivery mode:** individual; face to face  **Setting:** hospital | Standard care | 3 months/Baseline-3-6 months (15.7%) | VO_2peak_: significantly increased in the exercise group;  LVEF, GLS, peak HR: no significant difference between groups |
| Jones et al. (2014)/USA | **Diagnosis:** cancer with heart failure  **Cancer therapy:** anticancer therapy  **Sample size:**  N=90  EG=47  CG=43  **Mean age:**  EG=66±10  CG=66±11 | **Content:** aerobic training (treadmill or stationary cycle ergometer) and standardized usual care materials and physical activity recommendations  **No./length/frequency of session:** three 20 to 45-min weekly exercises at 60% to 70% of heart rate reserve (HRR) (initiated at 15 to 30 minutes per session at 60% HRR; after six sessions, the duration and intensity were increased to 30 to 35 minutes and 70% of HRR, respectively)  **Format/delivery mode:** group; face to face  **Setting:** hospital | Usual care (maintain usual exercise levels and standardized usual care materials and physical activity recommendations) | 3 months/Baseline-3-12 months (14.4%) | VO_2peak_ and HR: no significant difference between groups |
| Jones et al. (2013)/USA | **Diagnosis:** breast cancer  **Cancer therapy:** Neoadjuvant Chemotherapy (neoadjuvant doxorubicin and cyclophosphamide; or cyclophosphamide and adjuvant endocrine therapy)  **Sample size:**  N=20  EG=10  CG=10  **Mean age:**  EG=51±6  CG=46±11 | **Content:** aerobic exercise training (cycle ergometry)  **No./length/frequency of session:** three 20 to 45-min weekly exercises (intensity at 55% to 100% of VO_2peak_)  **Format/delivery mode:** individual; face to face  **Setting:** hospital | Maintain usual exercise levels | 3 months/Baseline-3 months (5%) | FMD: significantly improved in the exercise group; |
| Kerrigan et al. (2023)/USA | **Diagnosis:** breast cancer and leiomyosarcoma  **Cancer therapy:** chemotherapy (doxorubicin) or targeted therapy (trastuzumab)  **Sample size:**  N=22  EG=11  CG=11  **Mean age:**  EG=58±11  CG=52±13 | **Content:** cardiac rehabilitation (warm-up, interval training on treadmill, stationary cycle, elliptical trainer, etc., cool down), education lectures, and follow-up call)  **No./length/frequency of session:** two or three 40 to 50-min weekly exercises (5-min warm-up, 4-min high-intensity intervals at 71-90% heart rate reserve alternated by 3 min of moderate intensity at 60-70%, and 5-min cool down) and biweekly follow-up call  **Format/delivery mode:** individual; face to face  **Setting:** hospital | Follow physician instructions regarding care | 10 weeks/Baseline-10 weeks (24%) | VO_2peak_: significantly increased in the exercise group;  GLS, hs-cTnI, and peak HR: no significant difference between groups |
| Kim et al. (2006)/South Korea | **Diagnosis:** breast cancer  **Cancer therapy:** chemotherapy and/or radiotherapy  **Sample size:**  N=41  EG=22  CG=19  **Mean age:**  EG=51.3±6.7  CG=48.3±8.8 | **Content:** moderate-intensity aerobic exercise program (cycling, walking, jogging, or running on a treadmill or track; stress management consisted of relaxation training, cognitive behavioral modifications, sharing of cancer-related information and issues, and peer support; 7-day physical activity log; follow-up)  **No./length/frequency of session:** three 40-min weekly exercises (5-min warm-up, 30-min aerobic exercise at 60-70% of heart rate reserve and/or VO_2peak_ achieved on the Graded Exercise Test at baseline, 5-min cool down; reassessed and adjusted the participants’ heart rate responses weekly for first 3 weeks), one 90-min weekly stress management sessions, and biweekly follow-up  **Format/delivery mode:** individual and group; face to face  **Setting:** exercise facility within the School of Nursing and cardiac rehabilitation facility | 7-day physical activity log and follow-up | 2 months/Baseline-2-6 months (not reported) | VO_2peak_: significantly increased in the exercise group;  Resting HR, peak HR, and resting SBP: no significant difference between groups |
| Kirkham et al. (2018)/Canada | **Diagnosis:** breast cancer  **Cancer therapy:** chemotherapy (doxorubicin)  **Sample size:**  N=24  EG=13  CG=11  **Mean age:**  EG=51±9  CG=50±10 | **Content:** aerobic therapy (treadmill bout approximately 24h prior to each doxorubicin treatment; abstain from vigorous-intensity aerobic exercise for 72 h prior to, and 48 h after each treatment) and light or moderate-intensity exercise at any time  **No./length/frequency of session:** 45-min exercise (10-min warm-up, 30-min vigorous intensity of 70% heart rate reserve, and 5-min cool down)  **Format/delivery mode:** individual; face to face  **Setting:** not reported | Usual care (light or moderate-intensity exercise) | Not reported/Baseline-end of treatment (11%) | GLS, hs-cTnT, NT-proBNP, SBP, DBP, MAP, LVEF: no significant difference between groups;  Resting HR, stroke volume, and cardiac output: significantly improved in the exercise group |
| Kirkham et al. (2023)/Canada | **Diagnosis:** breast cancer  **Cancer therapy:** chemotherapy (trastuzumab and/or anthracycline)  **Sample size:**  N=74  EG=37  CG=37  **Mean age:**  EG=53±10.0  CG=52±9.0 | **Content:** Cardiac Rehabilitation Care Model (a guideline-based management plan, a personalized nutritional diagnosis and recommendations, and supervised moderate-intensity aerobic and resistance training)  **No./length/frequency of session:** two 60 to 90-minute weekly exercise sessions  **Format/delivery mode:** group; face to face  **Setting:** hospital | Usual care | 52 weeks/Baseline-24 weeks-52 weeks (7.8%) | LVEF, GLS, hs-cTnI, BNP, stroke volume, and VO_2peak_: no significant difference between groups |
| Lee et al. (2019)a/USA | **Diagnosis:** breast cancer  **Cancer therapy:** chemotherapy (doxorubicin and cyclophosphamide)  **Sample size:**  N=30  EG=15  CG=15  **Mean age:**  EG=49.1±7.9  CG=44.7±11.2 | **Content:** high intensity interval training (warm-up, high intensity interval training on the stationary bike, cool down)  **No./length/frequency of session:** three 30-min weekly exercises (5-min warm-up, 20-min high intensity interval training (7 times of a 1-min interval performed at 90% peak power output followed by a 2-min recovery interval performed at 10% PPO), 5-min cool down)  **Format/delivery mode:** individual; face to face  **Setting:** hospital | Non-exercise control (maintain current level of physical activity) | 2 months/Baseline-2 months (0%) | VO_2max_: no significant difference between groups |
| Lee et al. (2019)b/USA | **Diagnosis:** breast cancer  **Cancer therapy:** chemotherapy (doxorubicin and cyclophosphamide)  **Sample size:**  N=30  EG=15  CG=15  **Mean age:**  EG=49.1±7.9  CG=44.7±11.2 | **Content:** high intensity interval training (warm-up, high intensity interval training on the stationary bike, cool down)  **No./length/frequency of session:** three 30-min weekly exercises (5-min warm-up, 20-min high intensity interval training (7 times of a 1-min interval performed at 90% peak power output followed by a 2-min recovery interval performed at 10% PPO), 5-min cool down)  **Format/delivery mode:** individual; face to face  **Setting:** hospital | Non-exercise control (maintain current level of physical activity) | 2 months/Baseline-2 months (0%) | baPWV: significantly improved in the exercise group;  Resting SBP and resting DBP: no significant difference between groups |
| Mijwel et al. (2018)A/Sweden | **Diagnosis:** breast cancer  **Cancer therapy:** chemotherapy (anthracycline or taxane)  **Sample size:**  N=134  EG=74  CG=60  **Mean age:**  EG=52.7±10.3  CG**=**52.6±10.2 | **Content:** resistance and high-intensity interval training  **No./length/frequency of session:** two weekly exercises (resistance training: 8–12 repetitions at 75–80% of one repetition maximum targeting the major muscle groups; 3×3 min bouts of aerobic high intensity interval training on a cycle ergometer)  **Format/delivery mode:** individual; face to face  **Setting:** hospital | Usual care (written information about the exercise recommendations based on guidelines) | 4 months/Baseline-4 months (7.5%) | VO_2peak_: significantly decreased in the control group |
| Mijwel et al. (2018)B/Sweden | **Diagnosis:** breast cancer  **Cancer therapy:** chemotherapy (anthracycline or taxane)  **Sample size:**  N=132  EG=72  CG=60  **Mean age:**  EG=54.4±10.3  CG**=**52.6±10.2 | **Content:** moderate-intensity aerobic and high-intensity interval training  **No./length/frequency of session:** two weekly exercises (20-min moderate-intensity continuous aerobic exercise and 3×3 min bouts of aerobic high intensity interval training on a cycle ergometer)  **Format/delivery mode:** individual; face to face  **Setting:** hospital | Usual care (written information about the exercise recommendations based on guidelines) | 4 months/Baseline-4 months (9.3%) | VO_2peak_: significantly decreased in the control group |
| Northey et al. (2019)A/Australia | **Diagnosis:** breast cancer  **Cancer therapy:** chemotherapy and/or radiotherapy  **Sample size:**  N=12  EG=6  CG=6  **Mean age:**  EG=60.3±8.1  CG**=**61.5±7.8 | **Content****:** high-intensity interval training (warm-up, high-intensity interval training, and cool down on cycle ergometer)  **No./length/frequency of session:** three 20 to 30-min weekly exercises (5-min warm-up and cooldown at 50% of peak power; initially (week 1) completed four intervals lasting 30s with 2-min of active recovery between each; The number of intervals was increased by one each week until the target of seven intervals was achieved in week 4; The number of intervals were maintained at seven for the remainder of the intervention; increased the pedaling rate to between 95 and 115 revolutions per minute (RPM) and reached a heart rate above 90% of the maximum by the fourth interval)  **Format/delivery mode:** individual; face to face  **Setting:** not reported | Maintain current levels of physical activity and wait-list control | 3 months/Baseline-3 months (0%) | VO_2peak_: significantly increased in the exercise group;  MAP: no significant difference between groups |
| Northey et al. (2019)B/Australia | **Diagnosis:** breast cancer  **Cancer therapy:** chemotherapy and/or radiotherapy  **Sample size:**  N=11  EG=5  CG=6  **Mean age:**  EG=67.8±7.0  CG**=**61.5±7.8 | **Content:** moderate-intensity continuous training (warm-up, moderate-intensity continuous training, and cool down on cycle ergometer)  **No./length/frequency of session:** three 20 to 30-min weekly exercises (5-min warm-up and cooldown at 50% of peak power divided by a 20-min conditioning period completed at 55–65% of their peak power. The workload began at 55% of peak power and was adjusted within this range over 12 weeks to ensure their RPE remained between 9 and 13 on a modified Borg scale (6–20) during each session. Heart rate and RPE was measured at 5min intervals to monitor the participant’s response to the exercise)  **Format/delivery mode:** individual; face to face  **Setting:** not reported | Maintain current levels of physical activity and wait-list control | 3 months/Baseline-3 months (0%) | VO_2peak_ and MAP: no significant difference between groups |
| Schneider et al. (2023)/Switzerland | **Diagnosis:** breast cancer and lymphoma  **Cancer therapy:** chemotherapy (anthracycline)  **Sample size:**  N=57  EG=28  CG=29  **Mean age:**  EG=50 [38, 57]  CG**=**46 [38, 57] | **Content: s**upervised exercise training (24 supervised center-based, 12 non-supervised home-based exercise  sessions, counselling on PA, psychological aspects, nutrition, cardiovascular risk factors and pain management)  **No./length/frequency of session:** two 90-min weekly exercises  **Format/delivery mode:** group and individual; face to face and remote  **Setting:** hospital and home | Verbal recommendation of performing a minimum of 150 min of at least moderate exercise per week | 12 weeks/Baseline-End of treatment (week 8-14)-12 weeks follow up (week 20-26)/ (10.5%) | GLS, LVEF, E/A ratio, E/e' ratio, VO_2peak_, hs-CRP, hs-cTnT, NT-proBNP, SBP, and DBP: no significant difference between groups |
| Scott et al. (2018)/USA | **Diagnosis:** breast cancer  **Cancer therapy:** chemotherapy and/or radiotherapy and/or endocrine therapy  **Sample size:**  N=65  EG=33  CG=32  **Mean age:**  EG=52±10  CG**=**56±12 | **Content:** aerobic training (treadmill walking)  **No./length/frequency of session:** three weekly exercises (The intensity of each session alternated between four different dose intensities (i.e., 55%, 65%, 75%, and 80%) of maximal metabolic (MET) expenditure (i.e., VO_2peak_) based on participants’ workload)  **Format/delivery mode:** individual; face to face  **Setting:** hospital | Attention control (individualized stretching sessions) | 3 months/Baseline-3 months (23.1%) | VO_2peak_: no significant difference between groups |
| Segal et al. (2009)A/Canada | **Diagnosis:** prostate cancer  **Cancer therapy:** radiotherapy  **Sample size:**  N=81  EG=40  CG=41  **Mean age:**  EG=66.4±7.6  CG**=**65.3±7.6 | **Content:** resistance exercise training (warm-up and cool-down: light aerobic activity and stretching; resistance training: leg extension, leg curl, seated chest fly, latissimus pulldown, overhead press, triceps extension, leg curls, calf raises, low back extension, and modified curl-ups)  **No./length/frequency of session:** three 15 to 45-min (began at 15 minutes and increased by 5 minutes every 3 weeks until it reached 45 minutes) weekly exercises (5-min warm-up, two sets of eight to 12 repetitions of 10 different exercises at 60% to 70% of the estimated on-repetition maximum (1 RM); resistance was increased by 5 lb when participants completed more than 12 repetitions)  **Format/delivery mode:** individual; face to face  **Setting:** hospital | Usual care | 6 months/Baseline-3-6 months (12.3%) | VO_2peak_: significantly superior in the exercise group |
| Segal et al. (2009)B/Canada | **Diagnosis:** prostate cancer  **Cancer therapy:** radiotherapy  **Sample size:**  N=81  EG=40  CG=41  **Mean age:**  EG=66.2±6.8  CG**=**65.3±7.6 | **Content:** aerobic exercise training (exercise on the cycle ergometer, treadmill, or elliptical trainer)  **No./length/frequency of session:** three 15 to 45-min (began at 15 minutes and increased by 5 minutes every 3 weeks until it reached 45 minutes) weekly exercises (began at 50% to 60% of predetermined peak oxygen consumption (VO_2peak_) for weeks 1 to 4 and progress to 70% to 75% for weeks 5 to 24)  **Format/delivery mode:** individual; face to face  **Setting:** hospital | Usual care | 6 months/Baseline-3-6 months (4.9%) | VO_2peak_: no significant difference between groups |
| Siripanya et al. (2023)/Thailand | **Diagnosis:** breast cancer  **Cancer therapy:** chemotherapy (anthracycline)  **Sample size:**  N=30  EG=15  CG=15  **Mean age:**  EG=45.0±8.0  CG**=**44.9±8.0 | **Content:** Walking meditation exercise intervention (listen to a sound audio rhythm (MP3 recorder) at 25 beats/min, voiced “Budd” and “Dha; squeeze rubber balls in both hands rhythmically and simultaneously)  **No./length/frequency of session:** phase 1 (weeks 1-6): three 30-minute weekly walking exercises (at an intensity of 41%–50% of individual heart rate reserve; separated in 3 intervals, 10 min/interval with 3-min rest between the intervals); phase 2 (weeks 7-12): three 30-minute weekly walking exercises (at an intensity of 51%–60% of individual heart rate reserve; (separated in 3 intervals, 15 min/ interval with 3-min rest between the intervals)  **Format/delivery mode:** individual; face to face  **Setting:** home | Non-exercising control | 12 weeks/Baseline-12 weeks (26.7%) | VO_2peak_, FMD, hs-CRP, stroke volume, and cardiac output: significant difference between groups |
| Thorsen et al. (2005)/Norway | **Diagnosis:** malignant lymphomas and breast, gynecologic, or testicular cancer  **Cancer therapy:** chemotherapy (cyclophosphamide, methotrexate, and fluorouracil; or fluorouracil, or epirubicin, and cyclophosphamide) and surgery and radiotherapy  **Sample size:**  N=111  EG=59  CG=52  **Mean age:**  EG=39±8.4  CG**=**39.1±8.6 | **Content:**  home-based flexible training program (walking, cycling, strength training, water activities, aerobics, cross-country skiing, jogging, or ball games)  **No./length/frequency of session:** two 30-min weekly exercises (maintain intensity of a level between 13 and 15 based on Borg scale; or keep intensity levels at 60% to 70% of maximal heart rates; choice of activity and dose of training were readjusted every 2 weeks)  **Format/delivery mode:** individual; face to face  **Setting:** hospital | Usual care | 14 weeks/Baseline-14 weeks (20.1%) | VO_2peak_: significantly superior in the exercise group |
| Viamonte et al. (2023)/Portugal | **Diagnosis:** cancer survivors with high cardiovascular risk  **Cancer therapy:** high-dose anthracycline or high-dose radiotherapy, low-dose anthracycline, or anti-ERBB2 drugs  **Sample size:**  N=80  EG=40  CG=40  **Mean age:**  EG=54.5±13.5  CG**=**53.8±10.7 | **Content:** cardiac rehabilitation program (10-minute warm up, 30 to 40-minute aerobic exercise, 10 to 15-minute dynamic resistance exercise, and 5 to 10-minute cool down; individual dietary plan; psychological management and lifestyle behavior change; health education)  **No./length/frequency of session:** two weekly exercises, one individual dietary plan, a weekly group session on psychological management and lifestyle behavior change; a monthly health educational group session  **Format/delivery mode:** group; face to face  **Setting:** hospital | community-based exercise training program (nutritional and psychosocial management; two combined exercise sessions per week) | 8 weeks/Baseline-8 weeks (6.3%) | VO_2peak_, resting SBP, resting DBP, and resting HR: significantly improved in the exercise group  hs-CRP: no significant difference between groups |
| Windsor et al. (2004)/UK | **Diagnosis:** prostate carcinoma  **Cancer therapy:** radiotherapy  **Sample size:**  N=65  EG=32  CG=33  **Mean age:**  EG=68.3±0.9  CG**=**69.3±1.3 | **Content:** aerobic exercise (walking)  **No./length/frequency of session:** three 30-min weekly exercises (at a target heart rate of 60% to 70% calculated maximum heart rate)  **Format/delivery mode:** individual; face to face  **Setting:** hospital | Perform normal activities | 1 month/Baeline-1 month (1.5%) | Resting HR: no significant difference between groups |
| Yen et al. (2019)/Taiwan, China | **Diagnosis:** head and neck cancer  **Cancer therapy:** chemotherapy (aldesleukin or cisplatin or carboplatin or gemcitabine or medroxyprogesterone or uracil-Tegafur) or targeted therapy (cetuximab)  **Sample size:**  N=72  EG=34  CG=38  **Mean age:**  EG=52±10.7  CG**=**54.8±9.7 | **Content:** multimodal exercise (warm-up: walking or stepping with simple limb movements; aerobic exercise and resistance exercise (shoulder flexion, extension, abduction, elbow flexion and exercise, hip flexion, extension, abduction, knee flexion, and extension))  **No./length/frequency of session:** three 40 to 50-min weekly exercises (moderate intensity of the maximum HR ranging between 60% and 70%; 5-min warm-up, 30-min aerobic exercise training, 5-min cool down; resistance exercise consisted of ten repetitions for one set, three sets per training, with one repetition lasting 3 s.)  **Format/delivery mode:** individual; face to face  **Setting:** hospital | General health education | 2 months/Baseline-2 months (14.3%) | Resting HR, peak HR, resting SBP, resting DBP, peak SBP, and peak DBP: significantly improved in the exercise group |
| Zhou et al. (2018)/Mainland China | **Diagnosis:** nasopharyngeal carcinoma  **Cancer therapy:** chemoradiotherapy  **Sample size:**  N=114  EG=57  CG=57  **Mean age:**  EG=not reported  CG**=**not reported | **Content:** Tai Chi exercise (simplified 24-form Yang style; meditation and breathing techniques)  **No./length/frequency of session:** five one-hour weekly exercises (10-min warm-up, 30-min Tai Chi exercises, 10-min meditation and breathing techniques, and 10-min relaxation)  **Format/delivery mode:**  individual; face to face  **Setting:** hospital | Usual care | Not reported/Baseline-end of treatment (27.2%) | HRV: significantly improved in the exercise group |

Note: LVEF, left ventricular ejection fraction; GLS, global longitudinal strain; HR, heart rate; SBP, systolic blood pressure; DBP, diastolic blood pressure; hs-CRP, high-sensitivity C-reactive protein; hs-cTnI, high-sensitivity cardiac troponin I; hs-cTnT, high-sensitivity cardiac troponin T; BNP: brain natriuretic peptide; NT-proBNP, N-terminal prohormone of brain natriuretic peptide; MAP, mean arterial pressure; HRV, heart rate variability; FMD, flow-mediated dilation; baPWV, brachial–ankle pulse wave velocity.


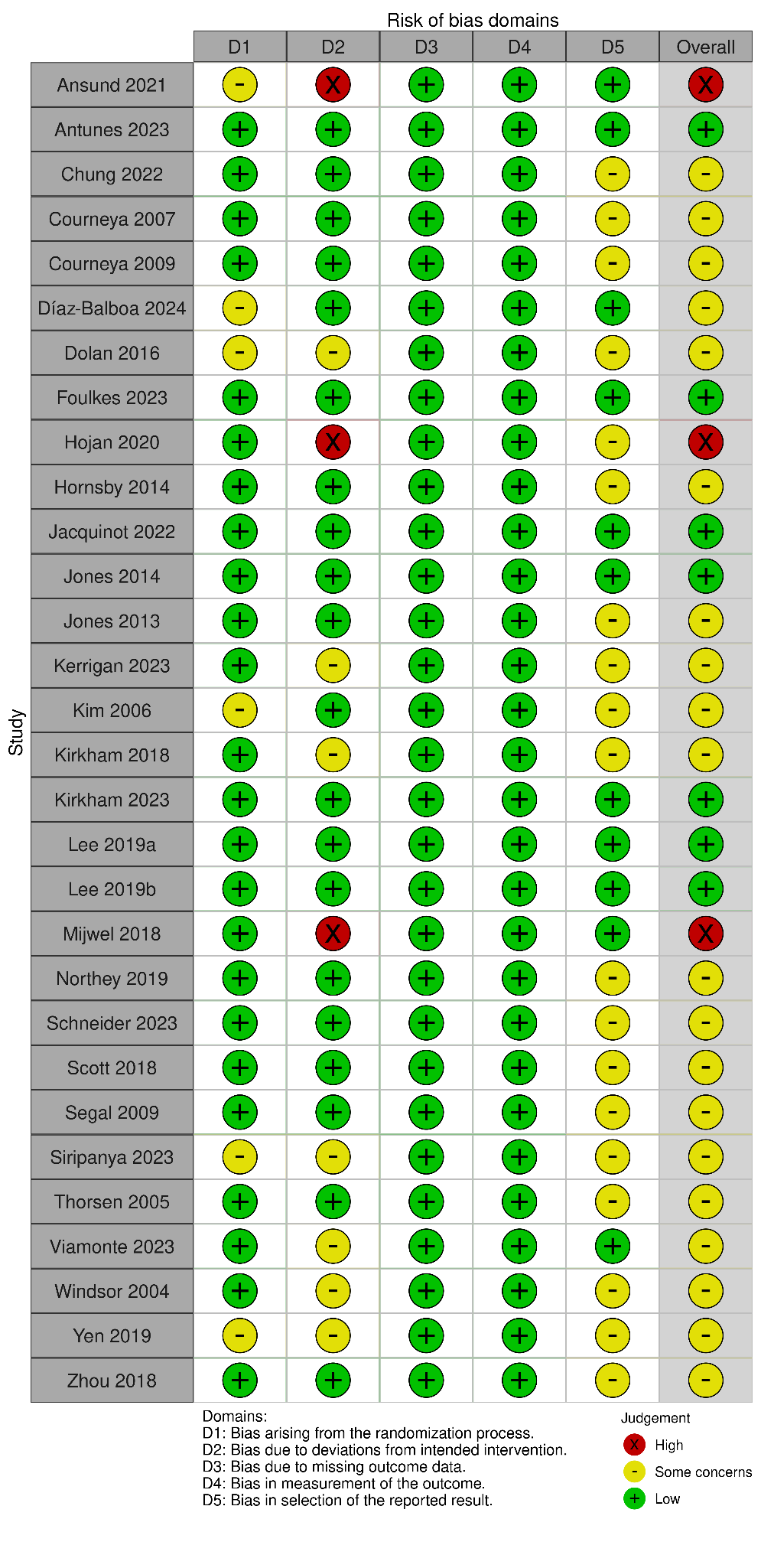


Appendix 4 The results of the quality appraisal

**Appendix 5 Summary of i-CONTENT assessment**

| Study | Patient selection | Dosage of the Exercise Program | Type of the Exercise Program | Qualified Supervisor (if applicable) | Type and Timing of Outcome Assessment | Safety of the Exercise Program | Adherence to the Exercise Program |
| --- | --- | --- | --- | --- | --- | --- | --- |
| Ansund et al. (2021)A | Low risk | Low risk | Low risk | Probably done | Low risk | Probably done | Probably not done |
| Ansund et al. (2021)B | Low risk | Low risk | Low risk | Probably done | Low risk | Probably done | Probably not done |
| Antunes et al. (2023) | Low risk | Low risk | Low risk | Low risk | Low risk | Low risk | Low risk |
| Chung et al. (2022) | Low risk | Low risk | Low risk | Low risk | Low risk | Low risk | Low risk |
| Courneya et al. (2007)A | Low risk | Low risk | Low risk | Probably done | Low risk | Low risk | Low risk |
| Courneya et al. (2007)B | Low risk | Low risk | Low risk | Probably done | Low risk | Low risk | Low risk |
| Courneya et al. (2009) | Low risk | Low risk | Low risk | Probably done | Low risk | Low risk | Low risk |
| Díaz-Balboa et al. (2024) | Low risk | Low risk | Low risk | Low risk | Low risk | Low risk | Low risk |
| Dolan et al. (2016)A | Low risk | Low risk | Low risk | Probably done | Low risk | Low risk | Low risk |
| Dolan et al. (2016)B | Low risk | Low risk | Low risk | Probably done | Low risk | Low risk | Low risk |
| Foulkes et al. (2023) | Low risk | Low risk | Low risk | Low risk | Low risk | Low risk | Low risk |
| Hojan et al. (2020) | Low risk | Low risk | Low risk | Low risk | Low risk | Low risk | Low risk |
| Hornsby et al. (2014) | Low risk | Low risk | Low risk | Probably done | Low risk | Low risk | Low risk |
| Jacquinot et al. (2022) | Low risk | Low risk | Low risk | Low risk | Low risk | Low risk | Probably done |
| Jones et al. (2014) | Low risk | Low risk | Low risk | Probably done | Low risk | Low risk | Probably done |
| Jones et al. (2013) | Low risk | Low risk | Low risk | Probably done | Low risk | Probably done | High risk |
| Kerrigan et al. (2023) | Low risk | Low risk | Low risk | Probably done | Low risk | Low risk | High risk |
| Kim et al. (2006) | Low risk | Low risk | Low risk | Probably done | Low risk | Low risk | Low risk |
| Kirkham et al. (2018) | Low risk | Low risk | Low risk | Probably done | Low risk | Low risk | Low risk |
| Kirkham et al. (2023) | Low risk | Low risk | Low risk | Low risk | Low risk | Probably done | Low risk |
| Lee et al. (2019)a | Low risk | Low risk | Low risk | Low risk | Low risk | Probably done | Low risk |
| Lee et al. (2019)b | Low risk | Low risk | Low risk | Low risk | Low risk | Probably done | Low risk |
| Mijwel et al. (2018)A | Low risk | Low risk | Low risk | Low risk | Low risk | Probably done | Probably done |
| Mijwel et al. (2018)B | Low risk | Low risk | Low risk | Low risk | Low risk | Probably done | Probably done |
| Northey et al. (2019)A | Low risk | Low risk | Low risk | Probably done | Low risk | Probably done | Low risk |
| Northey et al. (2019)B | Low risk | Low risk | Low risk | Probably done | Low risk | Probably done | Low risk |
| Schneider et al. (2023) | Low risk | Low risk | Low risk | Probably done | Low risk | Probably done | High risk |
| Scott et al. (2018) | Low risk | Low risk | Low risk | Probably done | Low risk | High risk | High risk |
| Segal et al. (2009)A | Low risk | Low risk | Low risk | Low risk | Low risk | Low risk | Low risk |
| Segal et al. (2009)B | Low risk | Low risk | Low risk | Low risk | Low risk | Low risk | Low risk |
| Siripanya et al. (2023) | Low risk | Low risk | Low risk | Probably done | Low risk | Low risk | Probably done |
| Thorsen et al. (2005) | Low risk | Low risk | Low risk | Low risk | Low risk | Probably done | Low risk |
| Viamonte et al. (2023) | Low risk | Low risk | Low risk | Low risk | Low risk | Low risk | Low risk |
| Windsor et al. (2004) | Low risk | Low risk | Low risk | Low risk | Low risk | Low risk | Probably done |
| Yen et al. (2019) | Low risk | Low risk | Low risk | Probably done | Low risk | Probably done | Probably done |
| Zhou et al. (2018) | Low risk | Low risk | Low risk | Low risk | Low risk | Probably done | Probably done |


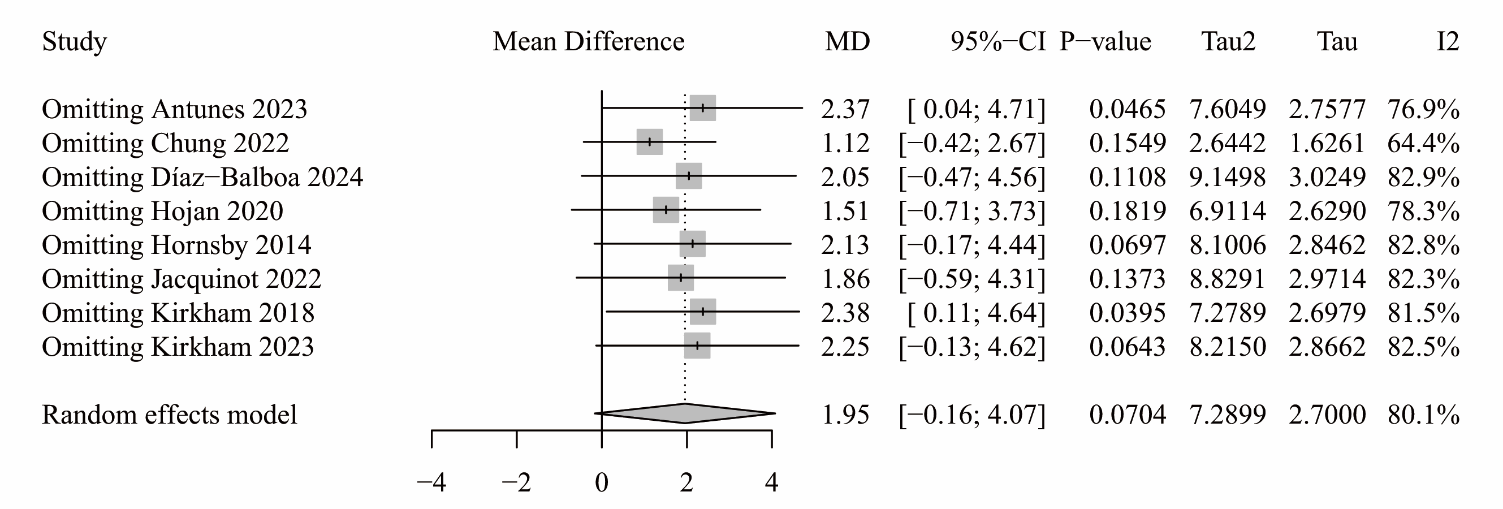


Appendix 6 Sensitivity analysis for the effects of exercise-based interventions on LVEF


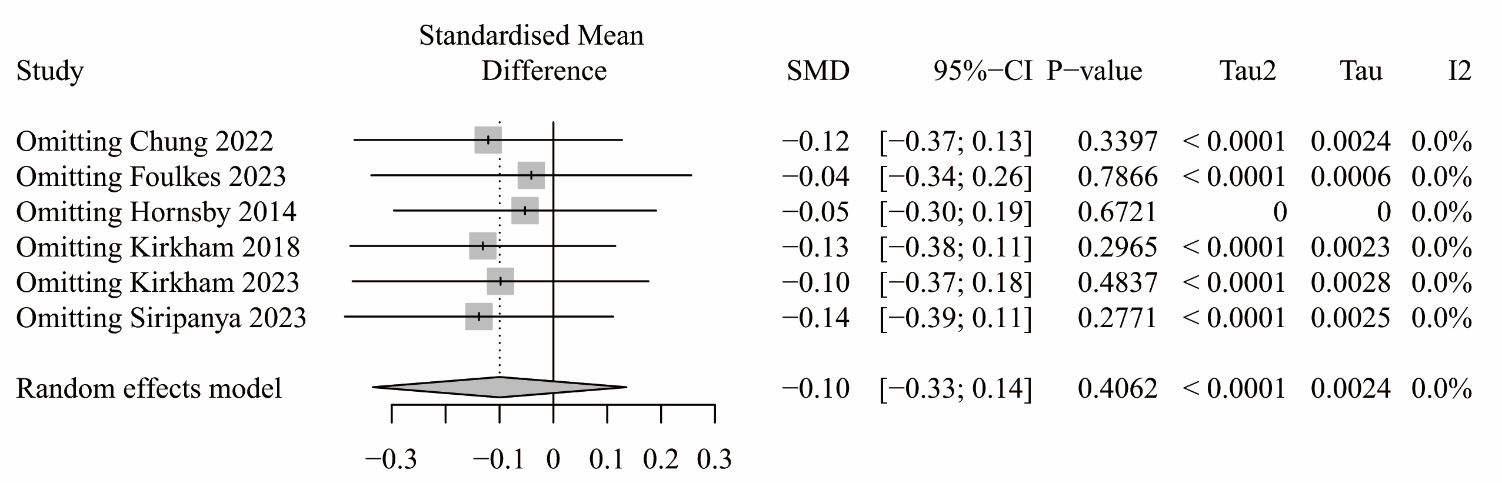


Appendix 7 Sensitivity analysis for the effects of exercise-based interventions on stroke volume


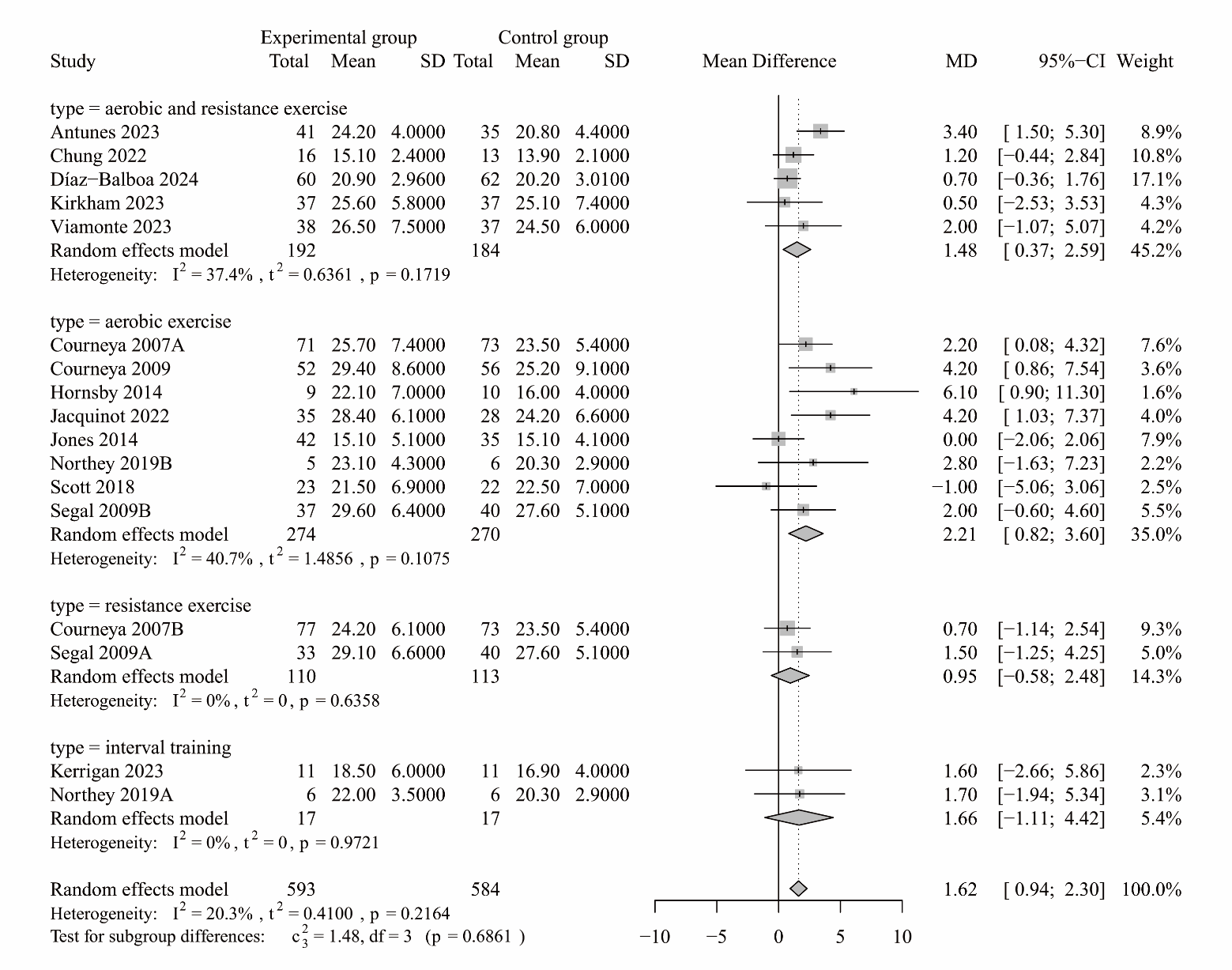


Appendix 8 Subgroup analysis for the effects of exercise-based interventions on VO_2peak_


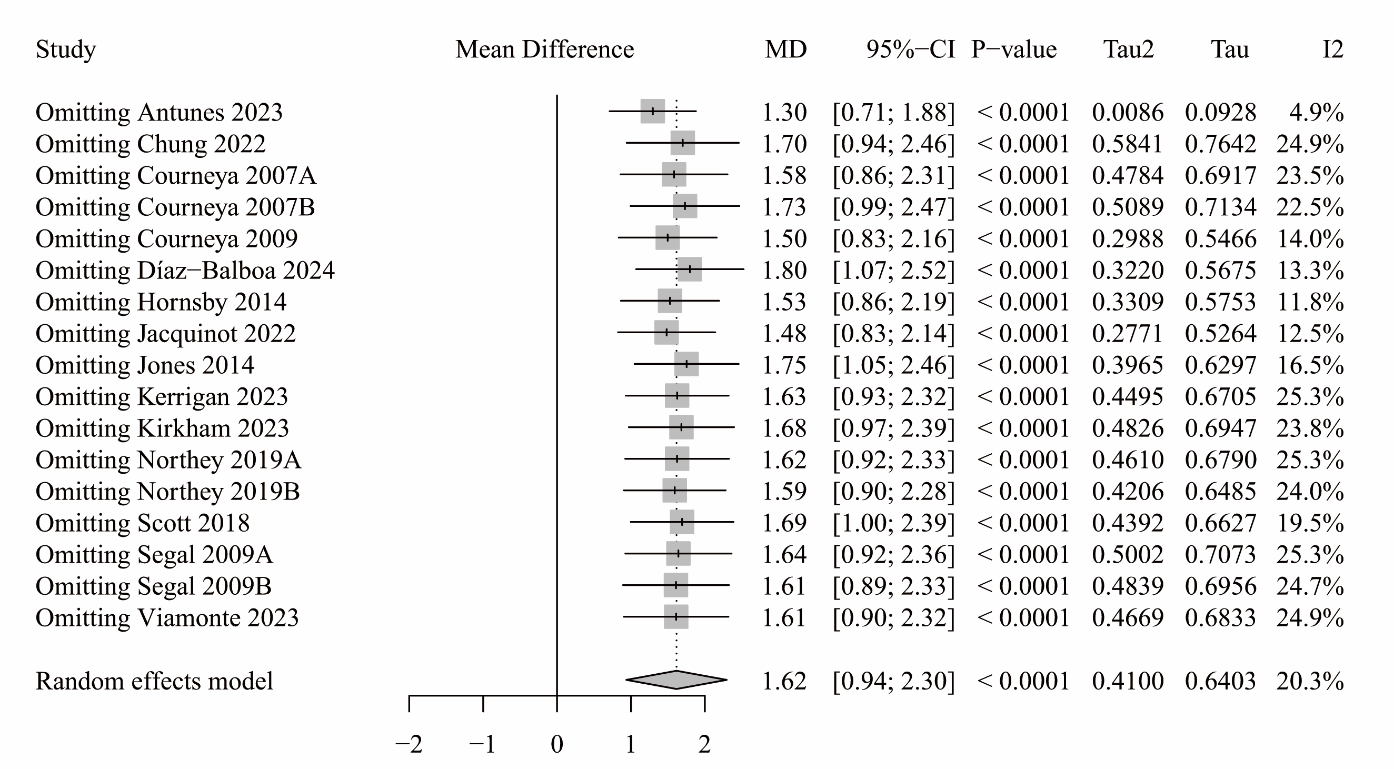


Appendix 9 Sensitivity analysis for the effects of exercise-based interventions on VO_2peak_


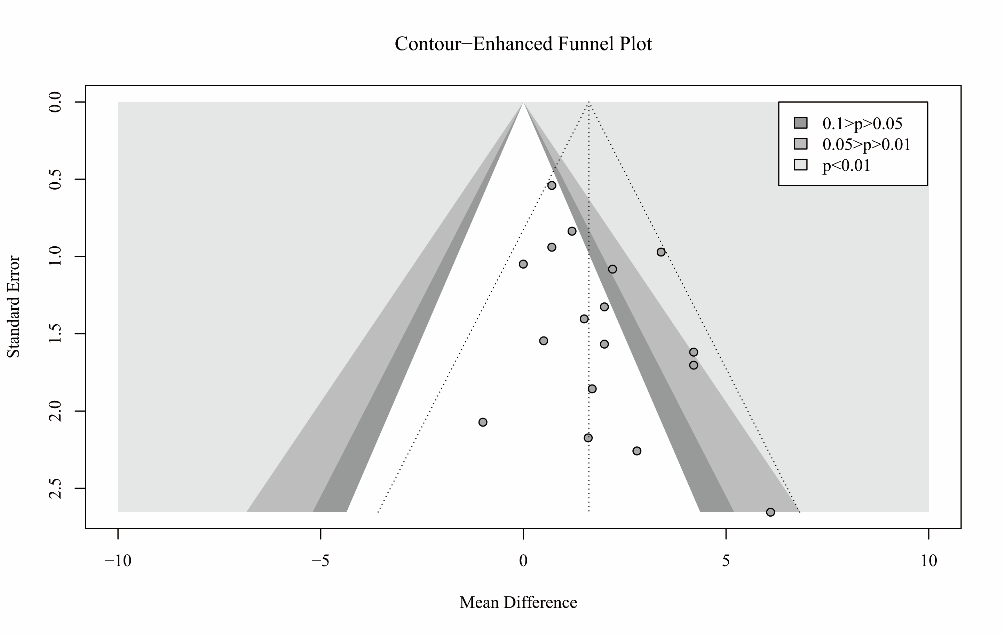


Appendix 10 Funnel plots for the effects of exercise-based interventions on VO_2peak_


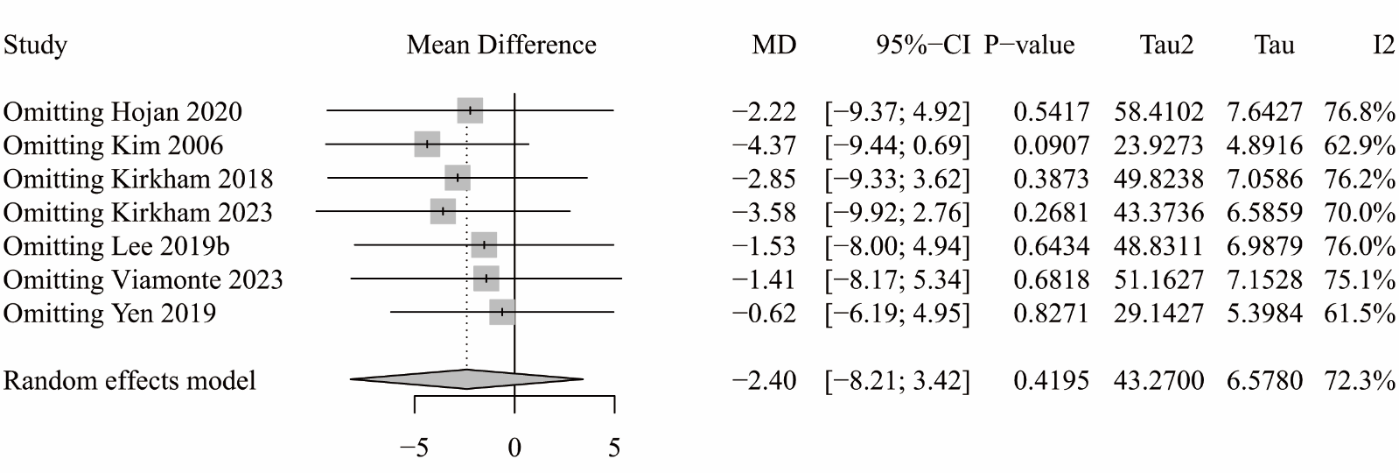


Appendix 11 Sensitivity analysis for the effects of exercise-based interventions on resting SBP


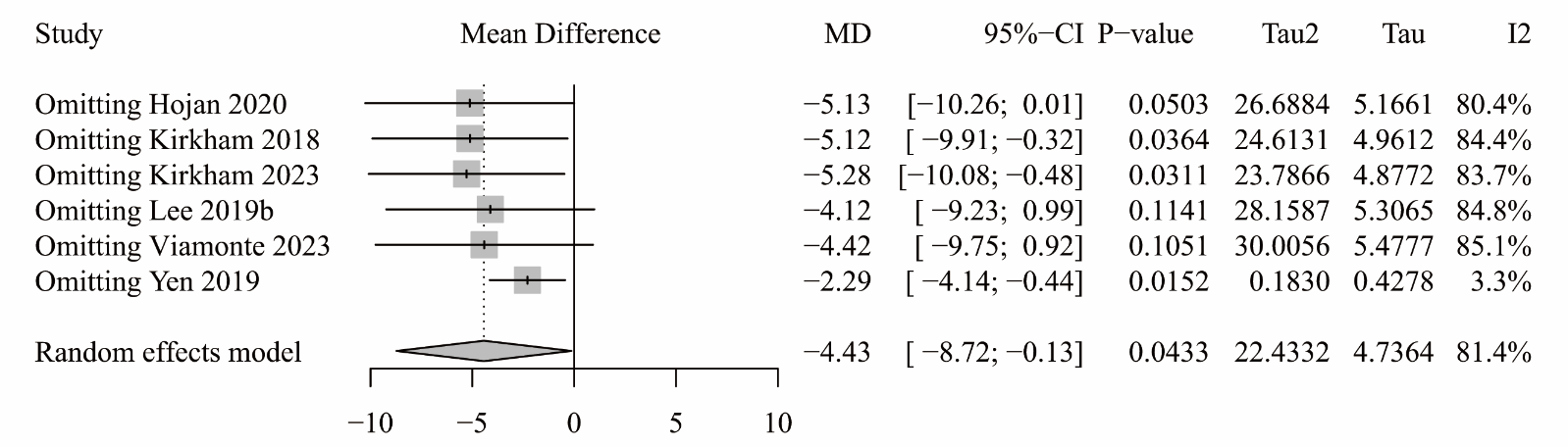


Appendix 12 Sensitivity analysis for the effects of exercise-based interventions on resting DBP


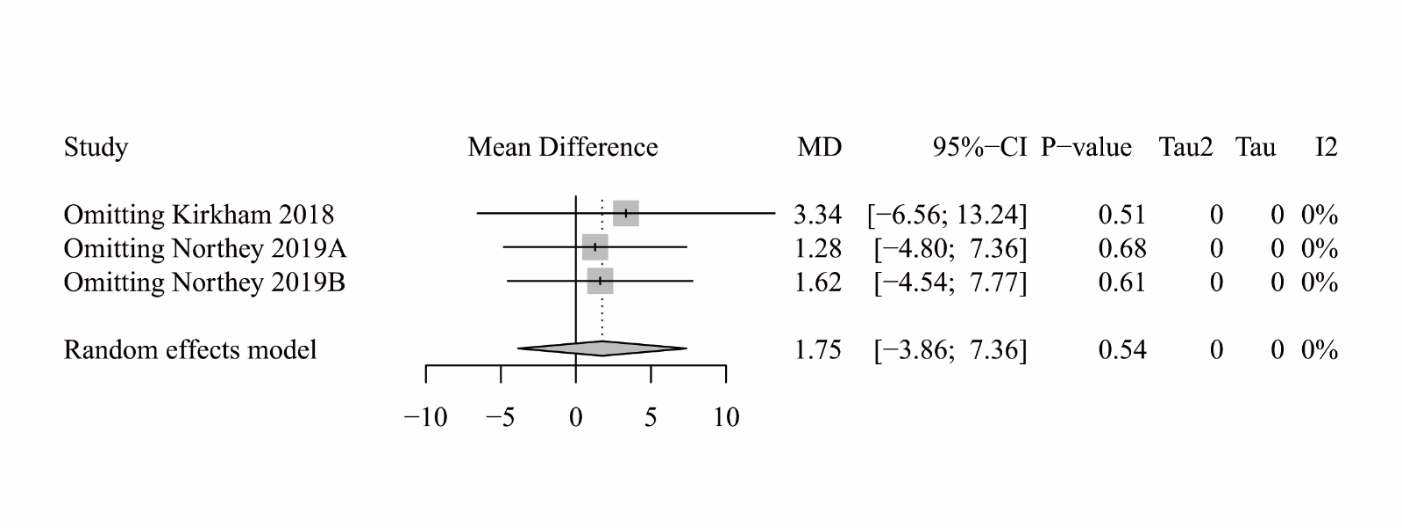


Appendix 13 Sensitivity analysis for the effects of exercise-based interventions on resting MAP


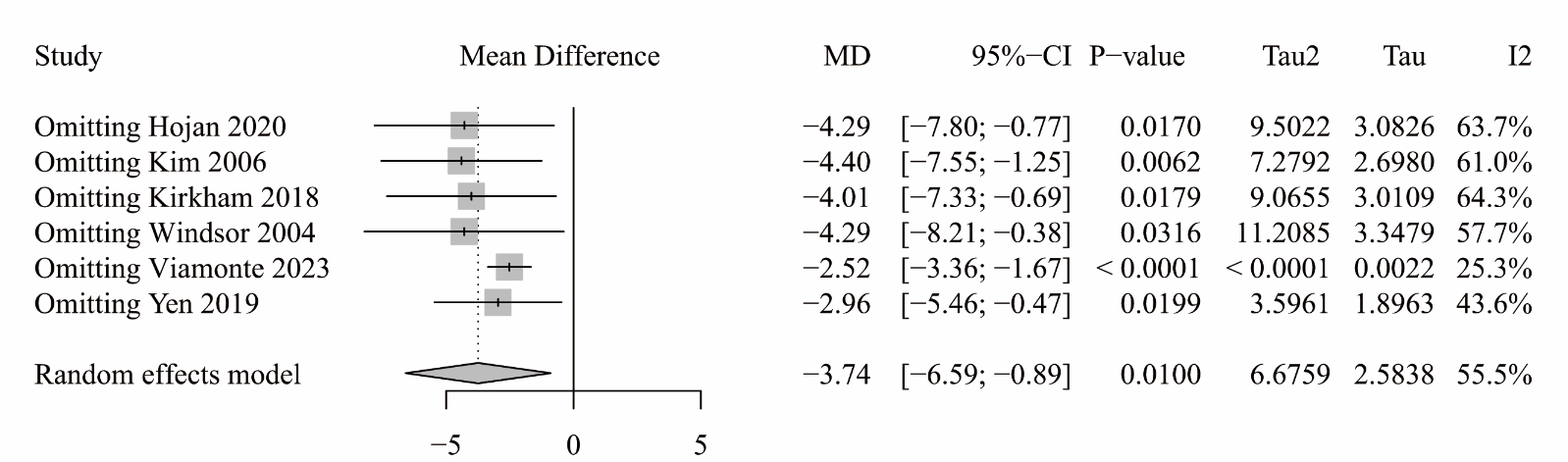


Appendix 14 Sensitivity analysis for the effects of exercise-based interventions on resting HR

| **Appendix 15 Summary of findings:** | | | | | | |
| --- | --- | --- | --- | --- | --- | --- |
| Outcomes | **Anticipated absolute effects^*^** (95% CI) | | Relative effect (95% CI) | № of participants (studies) | Certainty of the evidence (GRADE) | Comments |
|  | **Risk with control** | **Risk with exercise** |  |  |  |  |
| LVEF |  | MD **1.95 higher** (0.16 lower to 4.07 higher) | - | 482 (8 RCTs) | ⨁⨁⨁◯ Moderate^a^ |  |
| GLS | All the studies indicated that exercise-based interventions did not exert beneficial effects on GLS compared to the controls. | |  | 539 (8 RCTs) | ⨁⨁⨁◯ Moderate^a^ |  |
| Cardiac output | All the studies indicated that exercise-based interventions did not exert beneficial effects on cardiac output compared to the controls. | |  | 74 (3 RCTs) | ⨁⨁⨁◯ Moderate^a^ |  |
| Stroke volume | - | SMD **0.1 SD lower** (0.33 lower to 0.14 higher) | - | 281 (6 RCTs) | ⨁⨁⨁◯ Moderate^a^ |  |
| E/A ratio | One study indicated that exercise-based interventions did not exert beneficial effects on the E/A ratio compared to the controls, while the others found no significant difference between groups. | |  | 237 (4 RCTs) | ⨁⨁⨁◯ Moderate^a^ |  |
| E/e’ ratio | All the studies indicated that exercise-based interventions did not exert beneficial effects on the E/e’ ratio compared to the controls. | |  | 161 (2 RCTs) | ⨁⨁⨁◯ Moderate^a^ |  |
| VO_2peak_ |  | MD **1.62 higher** (0.94 higher to 2.3 higher) | - | 1177 (17 RCTs) | ⨁⨁⨁⨁ High |  |
| VO_2max_ | Only one study indicated that exercise-based interventions exert beneficial effects on VO2max compared to the controls. | |  | 141 (2 RCTs) | ⨁⨁⨁◯ Moderate^a^ |  |
| NT-proBNP | Only one study indicated that exercise-based interventions exert beneficial effects on NT-proBNP compared to the controls at 12 months, while the others found no significant difference between groups. | |  | 411 (6 RCTs) | ⨁⨁⨁⨁ High |  |
| BNP | One study indicated that exercise-based interventions did not exert beneficial effects on the BNP compared to the controls. | |  | 74 (1 RCT) | ⨁⨁⨁◯ Moderate^a^ |  |
| hs-cTnT | All the studies indicated that exercise-based interventions did not exert beneficial effects on the hs-cTnT compared to the controls. | |  | 289 (5 RCTs) | ⨁⨁⨁◯ Moderate^a^ |  |
| hs-cTnI | Only one study indicated that exercise-based interventions exert beneficial effects on hs-cTnI compared to the controls, while the others found no significant difference between groups. | |  | 322 (4 RCTs) | ⨁⨁⨁◯ Moderate^a^ |  |
| hs-CRP | Only one study indicated that exercise-based interventions exert beneficial effects on hs-CRP compared to the controls, while the others found no significant difference between groups. | |  | 214 (4 RCTs) | ⨁⨁⨁◯ Moderate^a^ |  |
| FMD | Only one study indicated that exercise-based interventions exert beneficial effects on FMD compared to the controls, while the other study found no significant difference between groups. | |  | 50 (2 RCTs) | ⨁⨁⨁◯ Moderate^a^ |  |
| baPWV | Only one study indicated that exercise-based interventions exert beneficial effects on baPWV compared to the controls, while the other study found no significant difference between groups. | |  | 60 (2 RCTs) | ⨁⨁⨁◯ Moderate^a^ |  |
| Resting SBP |  | MD **2.4 lower** (8.21 lower to 3.42 higher) | - | 363 (7 RCTs) | ⨁⨁⨁◯ Moderate^a^ |  |
| Resting DBP |  | MD **4.43 lower** (8.72 lower to 0.13 lower) | - | 322 (6 RCTs) | ⨁⨁◯◯ Low^a,b^ |  |
| Peak SBP | All the studies indicated that exercise-based interventions did not exert beneficial effects on the peak SBP compared to the controls. | |  | 176 (2 RCTs) | ⨁⨁⨁◯ Moderate^a^ |  |
| Peak DBP | All the studies indicated that exercise-based interventions did not exert beneficial effects on the peak DBP compared to the controls. | |  | 176 (2 RCTs) | ⨁⨁⨁◯ Moderate^a^ |  |
| MAP |  | MD **1.75 higher** (3.86 lower to 7.36 higher) | - | 46 (3 RCTs) | ⨁⨁⨁◯ Moderate^a^ |  |
| Resting HR |  | MD **3.74 lower** (6.59 lower to 0.89 lower) | - | 324 (6 RCTs) | ⨁⨁⨁◯ Moderate^a^ |  |
| Peak HR | Only one study indicated that exercise-based interventions exert beneficial effects on peak HR compared to the controls, while the others found no significant difference between groups. | |  | 406 (6 RCTs) | ⨁⨁⨁◯ Moderate^a^ |  |
| HRV | A study indicated that exercise-based interventions exert beneficial effects on peak HRV compared to the controls. | |  | 114 (1 RCT) | ⨁⨁⨁◯ Moderate^a^ |  |
| ***The risk in the intervention group** (and its 95% confidence interval) is based on the assumed risk in the comparison group and the **relative effect** of the intervention (and its 95% CI). **CI:** confidence interval; **MD:** mean difference; **SMD:** standardised mean difference; **LVEF:** left ventricular ejection fraction; **GLS;** global longitudinal strain; **HR:** heart rate; **SBP:** systolic blood pressure; **DBP:** diastolic blood pressure; **hs-CRP:** high-sensitivity C-reactive protein; **hs-cTnI:** high-sensitivity cardiac troponin I; **hs-cTnT:** high-sensitivity cardiac troponin T; **BNP:** brain natriuretic peptide; **NT-proBNP:** N-terminal prohormone of brain natriuretic peptide; **MAP:** mean arterial pressure; **HRV:** heart rate variability; **FMD:** flow-mediated dilation; **baPWV:** brachial–ankle pulse wave velocity. | | | | | | |
| **GRADE Working Group grades of evidence** **High certainty:** we are very confident that the true effect lies close to that of the estimate of the effect. **Moderate certainty:** we are moderately confident in the effect estimate: the true effect is likely to be close to the estimate of the effect, but there is a possibility that it is substantially different. **Low certainty:** our confidence in the effect estimate is limited: the true effect may be substantially different from the estimate of the effect. **Very low certainty:** we have very little confidence in the effect estimate: the true effect is likely to be substantially different from the estimate of effect.  a: downgrade one level for the imprecision due to the total sample size does not meet the rules of thumb (at least 400 participants) and/or narrative synthesis was conducted.  b: downgrade one level for the inconsistency due to considerable heterogeneity (I2 > 75%). | | | | | | |
